# Perceptions, attitudes, behaviours and barriers towards obesity among people with obesity and health care professionals in Indonesia: An exploratory online survey

**DOI:** 10.1371/journal.pone.0350857

**Published:** 2026-06-04

**Authors:** Sidartawan Soegondo, Gaga Irawan Nugraha, Farid Kurniawan, Ana Asmara Jannati, Dicky L. Tahapary

**Affiliations:** 1 Division of Endocrinology, Metabolism, and Diabetes, Department of Internal Medicine, Dr. Cipto Mangunkusumo National General Hospital/Faculty of Medicine Universitas Indonesia, Jakarta, Indonesia; 2 Indonesia Diabetes Institute, Jakarta, Indonesia; 3 Diabetes Connection and Care, EKA Hospital, Banten, Indonesia; 4 Indonesian Society for the Study of Obesity, Jakarta, Indonesia; 5 Department of Biomedical Science, Faculty of Medicine, Universitas Padjajaran, West Java, Indonesia; 6 Metabolic Disorder, Cardiovascular and Aging Research Cluster, The Indonesian Medical Education and Research Institute, Faculty of Medicine Universitas Indonesia, Jakarta, Indonesia; 7 Novo Nordisk, Jakarta, Indonesia; University of Tabuk, SAUDI ARABIA

## Abstract

**Background:**

The Awareness, Care, and Treatment In Obesity maNagement Asia Pacific (ACTION APAC) online survey identified perceptions, attitudes, and barriers to effective obesity care among People with Obesity (PwO) and Health Care Professionals (HCP) across nine APAC countries. Here, we present findings from Indonesia.

**Methods:**

This was a cross-sectional, observational, descriptive survey in PwO (≥18-year-old) with self-reported body mass index of ≥25 kg/m^2^ and HCPs who spent ≥50% of their time in direct patient care. The survey was conducted between 20 April, 2022 to 11 May, 2022. The questionnaires were approved by the institutional review board as per local regulations.

**Findings:**

A total of 1,000 PwO and 200 HCPs completed the survey. Notable differences were observed among PwO and HCPs in acknowledging obesity as a chronic disease (54% PwO and 90% HCPs), considering weight loss as PwO responsibility (91% PwO and 17% HCPs) and in agreeing that PwO were motivated to lose weight (76% PwO and 50% HCPs). Almost, two-thirds (67%) of PwO perceived themselves as either overweight or normal weight while only 30% discussed weight with their HCPs in the past five years. Financial concerns (45%) and assuming self-responsibility for weight loss (43%) were cited as the top reasons for not discussing. Only 53% HCPs initiated weight conversations, as they believed that PwO were either not motivated (55%) or not able to lose weight (51%). When discussed, most (68%) HCPs recommended lifestyle changes.

**Interpretation:**

Our study identified gaps in understanding obesity as a disease and its management among PwO and HCPs, highlighting a need for increased awareness to improve obesity care in Indonesia.

## Introduction

Obesity is a chronic disease with complex pathophysiology. By 2035, it is projected that four billion people globally will be living with overweight and obesity, almost a two-fold increase from 2020 [1]. Similarly, in the South-East Asia region, the prevalence of obesity is expected to increase two-fold from 2020 to 2035 (4% to 10% in men and 8% to 16% in women) [1]. Traditionally, obesity was more prevalent in high-income countries. However, in the recent past the trends indicate that people from middle-, and low-income countries are also affected and are contributing largely to the increasing prevalence [2]. Additionally, the prevalence is higher in urban areas than in rural [2].

Indonesia, one of the largest economies and a highly populated country in the Asia-Pacific region, is classified as a lower-middle-income country. As of 2018, over one-third of Indonesian adults (64·4 million) were living with overweight or obesity [3]. By 2035, it is projected that 22% of Indonesian adults will be living with obesity, with an estimated annual increase of 5·8% [1]. This rise may be attributed to lifestyle changes driven by cultural shifts, rapid urbanization, and increased digitalization leading to sedentary behaviours [4]. Additionally, inadequate management approaches and varying definitions of obesity cut-off values may also have contributed to the growing prevalence [5]. Owing to its complex pathophysiology, management of obesity warrants a holistic approach. Understanding the dynamics between people with obesity (PwO) and healthcare professionals (HCPs) about the disease is crucial to foster a collaborative HCP-patient relationship, essential for delivering optimal care and supporting PwO in their journey to achieve optimal weight. With the rising obesity prevalence in Indonesia, it is a priority to ensure that PwO receive effective obesity care tailored to the country’s unique context.

There is a need for a comprehensive nationwide study in Indonesia to understand the awareness and knowledge of obesity management from the perspectives of PwO and HCP to identify and bridge the gaps, thereby improving obesity treatment strategies. The ACTION (Awareness, Care, and Treatment in Obesity maNagement) APAC, study was conducted to identify perceptions, attitudes and behaviors toward obesity and its management among both PwO and HCPs, and the potential barriers to effective obesity care across nine Asia-Pacific (APAC) countries, including Indonesia.

The findings from the regional ACTION-APAC study have been reported previously [6]. However, considering the increasing prevalence and burden of obesity in Indonesia, coupled with the rapid cultural shifts, changing food habits, socio-cultural differences, demographic characteristics and geographical challenges, it is important to explore and understand the results from the ACTION APAC with specific reference to Indonesia. Here, the objective of this manuscript is to report the results of the Indonesian population from the ACTION-APAC survey of PwO and HCPs.

## Methods

### Study design and setting

The comprehensive methodology of the ACTION APAC survey has been previously reported [6]. Briefly, this cross-sectional and non-interventional survey was conducted in nine countries (Bangladesh, India, Thailand, Malaysia, Pakistan, Philippines, Singapore, and Vietnam) including Indonesia, between April 14, 2022 and May 23, 2022 among PwO and HCPs. The survey was administered by KJT Group, Inc., Rochester, NY, USA through existing online databases/panels. Indonesian participants responded in either Indonesian or English, between April 20, 2022 and May 11, 2022.

### Study participants

Separate surveys were completed by PwO and HCPs, with all respondents providing informed consent electronically before study initiation. The questionnaires for PwO and HCP utilised in this online survey are provided in S1 and S2 Files, respectively. Eligible PwO were 18 years or older, residing in Indonesia with a current BMI of ≥25 kg/m^2^ calculated based on self‐reported height and weight. Individuals who had participated previously in the study, were actively involved in intense fitness programs, or had experienced a significant unintentional weight loss (WL) in the past six months were excluded. Eligible HCPs were physicians practicing in Indonesia whose primary specialty was general practice, family practice, internal medicine, or an appropriate specialty for the country. They were required to spend at least 50% of their time in patient medical management, have been in practice for more than two years, have seen at least 100 patients in the past month, and have seen at least ten patients in the past month who have obesity (BMI ≥ 25 kg/m^2^). An obesity specialist, defined as a physician who treats 50% or more of their patients for obesity. This specialty emerged naturally within the Indonesian study sample, as there were no specific targets or set quotas for obesity specialists. HCPs specializing in general, plastic or bariatric surgery were excluded.

Weights were applied to the PwO data to mitigate selection bias. To minimize PwO sampling bias and ensure generalizability, a stratified outbound sample was considered according to pre-determined demographics targets based on age, gender, household income, education, and region. These targets were established based on data from the 2011 International Standard Classification of Education (ISCED) and the US Census Bureau, International Data Base, and other publicly available information. Sample sizes were selected to ensure a balance between statistical power and recruitment feasibility. PwO sample sizes were aimed at achieving a 2–3% margin of error for a proportion estimates of 50%, with the margin of error calculated from a standard normal (Z-) distribution with z = 1·96, corresponding to a 95% confidence level.

### Study outcomes

The survey questions were based on a previous ACTION IO study [6], modified and refined for the APAC region including Indonesia, following extensive review and insights from a local steering committee of obesity experts (S1 and S2 File). Sixty-minute web-assisted pre-test interviews were conducted with six PwO and six HCPs (three each of primary care physician and specialists) in Indonesia to assess face validity prior to launch of the quantitative surveys. The detailed process of translation check and validation of survey are provided in S3 File. Outcome measures were assessed by a diverse list of items that included: overall health and wellbeing of PwO, WL discussions, attitudes towards obesity and its management, interactions with HCPs, impact of obesity and related stigma, health information sources, and socio-demographic characteristics. The responses were quantified through single and multiple item selection and numeric entries, reported as frequencies and percentages. A 5-point Likert scale indicating agreement, impact, or frequency was used to measure attitudes or opinions. The scale ranged from 1, “strongly disagree,” to 5, “strongly agree,” commonly employed for measuring agreement within the study.

### Data collection and analysis

The survey questionnaire items for both PwO and HCP were reviewed by the WIRB-Copernicus Group (WCG) Institutional Review Board and approved according to local regulations. The study and data collection were in agreement with all relevant country, federal, or state laws. The trial is registered with clinicaltrials.gov: NCT05250427 (February 22, 2022).

Data were collected using Decipher Survey Software (Focus Vision Worldwide Inc., Stamford, CT, USA) via a survey, administered using online platforms and supported through in-person interactions, or telephonic assistance based on the requirement. De-identified data were analysed by the KJT Group utilizing SPSS (version 23·0, IBM, Armonk, NY, USA), Stata (version IC 14·2, StataCorp LLC, College Station, TX, USA), and Excel (version 365, Microsoft, Redmond, WA, USA). The study adhered to the principles outlined in the Declaration of Helsinki and the European General Data Protection Regulation (GDPR).

Data were summarized descriptively using means, medians, and frequencies by respondent type. Categorical data were presented as counts and percentages. The analyses were performed using Q Research software for Windows 23 (A Division of Displyer, Inc., New South Wales, Australia).

## Results

### Baseline characteristics

A total of 1,000 PwO and 200 HCPs completed the survey. The PwO group had an equal gender distribution and a mean age of 34·3 years. The majority of PwO (68%) were in class I obesity category (BMI 25–29.9 kg/m^2^). Among HCPs 67% were male, with an average of 12·3 years in clinical practice, dedicating 70% of their time to direct patient care. The majority (76%) of HCPs identify themselves as obesity specialists. Furthermore, 78% of HCPs considered themselves as experts in obesity management and 87% reported having received advanced training in obesity care. Among PwO, 44% reported residing in urban areas, 39% in suburban areas, and 18% in rural areas or villages. Among HCPs, 75% are urban based, 25% suburban, with none from rural areas or villages (Table 1).

**Table 1:** Key demographics and baseline characteristics of the study participants.

Characteristic	PwO (n = 1,000)	HCPs (n = 200)
**Age, years, mean (SD)**	34·3 (10·7)	41.6 (7·2)
**Female, n (%)**	495 (50)	67 (34)
**Obesity class, n (%)** ^a^		
Class I (BMI 25–29·9 kg/m^2^)	675 (68)	--
Class II (BMI 30–34·9 kg/m^2^)	233 (23)	--
Class III (BMI 35–39·9 kg/m^2^)	62 (6)	--
Class IV (BMI ≥ 40 kg/m^2^)	30 (3)	--
**Setting, n (%)**		
Urban area	438 (44)	150 (75)
Suburban area	387 (39)	50 (25)
Rural area	175 (18)	0
**Income, n (%)**		
Less than 3,070,000 IDR	335 (34)	--
3,070,000–5,519,999 IDR	313 (31)	--
5,520,000–11,399,999 IDR	203 (20)	--
11,400,000–30,799,999 IDR	88 (9)	--
30,800,000 or more	25 (3)	--
Decline to answer	36 (4)	--
**Comorbidities, n (%)** ^b^		
None listed	419 (41)	--
High blood pressure	213 (22)	--
High cholesterol	149 (16)	--
Depression/anxiety	133 (16)	--
Eating disorder	131 (14)	--
Stomach or intestinal problems	144 (14)	--
Other conditions	75 (6)	--
Type 2 diabetes	60 (7)	--
**HCP practice category, n (%)**		
PCP		
Family practice	--	19 (10)
General practice,	--	50 (25)
Internal medicine	--	31 (16)
Specialist		
Endocrinology/ Diabetology	--	20 (10)
Cardiology	--	30 (15)
Gastroenterology	--	15 (8)
Nutrition specialist	--	15 (8)
Obstetrics/ Gynaecology	--	15 (8)
Bariatrics/ Obesity Medicine	--	5 (3)
Aesthetics medicine	--	0
Orthopaedist	--	0
**Years in practice, mean (SD)**	--	12·3 (6·5)
**Considered self an obesity expert, n (%)**	--	156 (78)
**Obesity specialist** ^**c**^ **, n (%)**	--	152 (76)

^a^BMI cut-offs are based on WHO Asia-Pacific region cut-off recommendations [7].

^b^Weighted data. Percentages do not add to 100 because respondents could select more than one condition.

^c^Obesity specialist is defined as a physician who reported seeing 50% or more patients specifically for obesity/weight management.

BMI, body mass index; HCPs, healthcare professionals; IDR, Indonesian Rupiah; PCP, primary care physician; PwO, people with obesity; SD, standard deviation.

### Attitudes about weight and obesity

**Fig 1:**
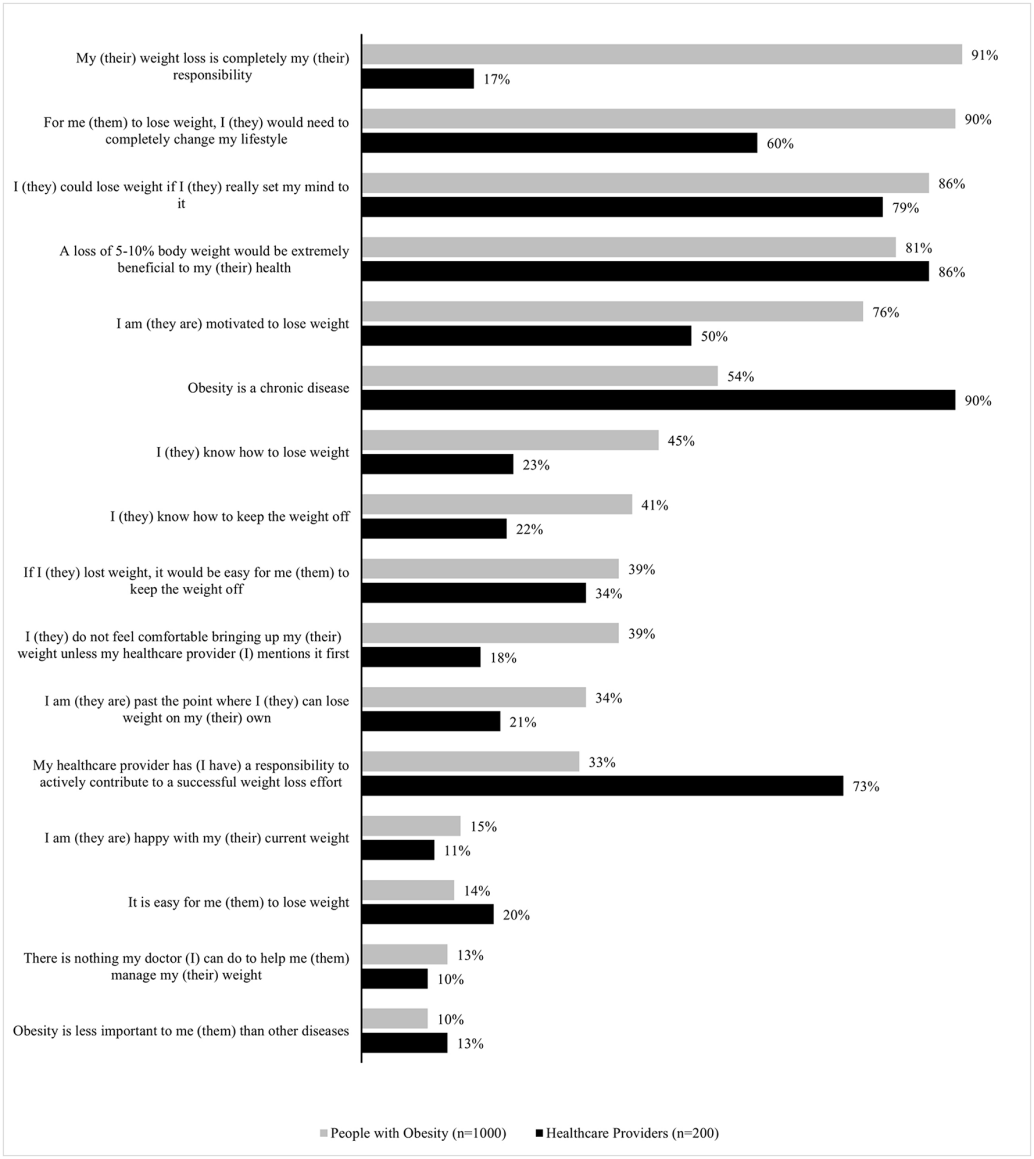
People with obesity (PwO) and healthcare professionals (HCPs) attitudes toward obesity. Percentage of PwO and HCPs who indicated their level of agreement was 5 on a 5-point scale where 1 meant ‘Do not agree at all’ and 5 meant ‘Completely agree’.

A total of 54% PwO and 90% of HCPs agreed that obesity is a chronic disease. Both PwO (81%) and HCPs (86%) believed that losing 5–10% of body weight is extremely beneficial to the health of PwO. However, most PwO (91%) considered weight loss as their responsibility, and 86% felt that they could lose weight if they set their mind to it but only 45% reported knowing how to lose weight. Most of PwO (76%) reported that they were motivated to lose weight, while only half of HCPs (50%) shared the same opinion. Notably, 15% of PwO reported being happy with their current weight and 11% of HCPs agreed (Fig 1). Both PwO (65%) and HCPs (89%) considered maintaining optimal weight as a priority for the country’s healthcare system. Also, 56% of PwO and 73% of HCPs regarded the cost of obesity treatment as a barrier to effective weight management.

### Motivators and barriers for weight loss

PwO reported their top motivators for losing weight as the desire to be more fit or to be in a better shape (56%), to feel better physically (52%) and wanting to be more confident (46%). However, HCPs believed that having general health concerns (56%), wanting to improve their job performance (53%) and wanting to be more confident or improve their self-esteem (47%) were the top motivators for PwO to lose weight (S1 Fig).

Both PwO and HCPs completely agreed that lack of exercise (79% PwO and 82% HCP), unhealthy eating habits (75% and 78%, respectively), high carbohydrate diet (70% and 71%, respectively), inability to control hunger (69% and 76%, respectively) and preference for unhealthy food (66% and 77%, respectively) were among the top barriers for PwO to lose weight. However, there were discrepancies between PwO and HCPs in their perception of additional barriers such as cost of weight management (53% PwO vs 44% HCP), mental health or emotional status (53% vs. 64%, respectively) and lack of understanding about the disease (42% vs 55%, respectively). Interestingly, 48% of PwO and 44% of HCPs believed that genetic predisposition was a barrier to WL (S2 Fig).

### Self-perception of current weight, serious weight loss attempts, and weight regain

**Fig 2:**
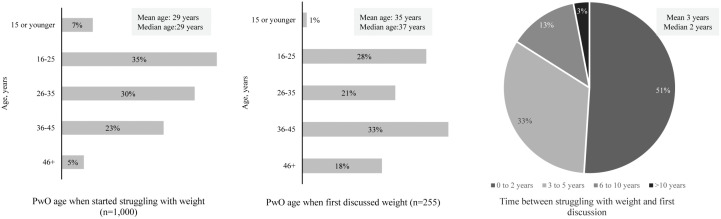
Age at which people with obesity (PwO) first struggled with weight and discussed their weight with their healthcare professional (HCP).

Although all PwO were living with obesity based on their self-reported weight and height and survey eligibility criteria, almost two in three (67%) perceived themselves as either overweight or of normal weight. PwO started struggling with weight at the mean age of 29 years, and it took them a mean of three years from the time they started struggling with weight to initiate a first discussion with their HCPs (Fig 2). On an average PwO made two serious WL attempts with 44% having made at least one serious WL attempt. Among PwO who experienced WL, 68% reported regaining weight after successfully maintaining it for at least six months. In comparision, HCPs reported that 50% of their PwO made a serious WL effort and that only 37% of those were successful. PwO cited not following the eating plan (55%), discontinuing exercise (49%) and difficulty staying motivated (40%) as the most common reasons for weight regain.

### Perceptions of weight stigma

Both PwO and HCPs believed that living with obesity made it harder for PwO to form a romantic relationship (63% PwO and 89% HCPs), get a job (65% and 68%, respectively) and be successful in the workplace (53% and 62%, respectively). Both groups also reported that being overweight somewhat or very negatively influenced how others’ perceived PwO in terms of being athletic (60% PwO and 87% HCPs), healthy (64% and 81%, respectively), or smart (24% and 73%, respectively).

### Discussion regarding weight between PwO and HCPs

**Fig 3:**
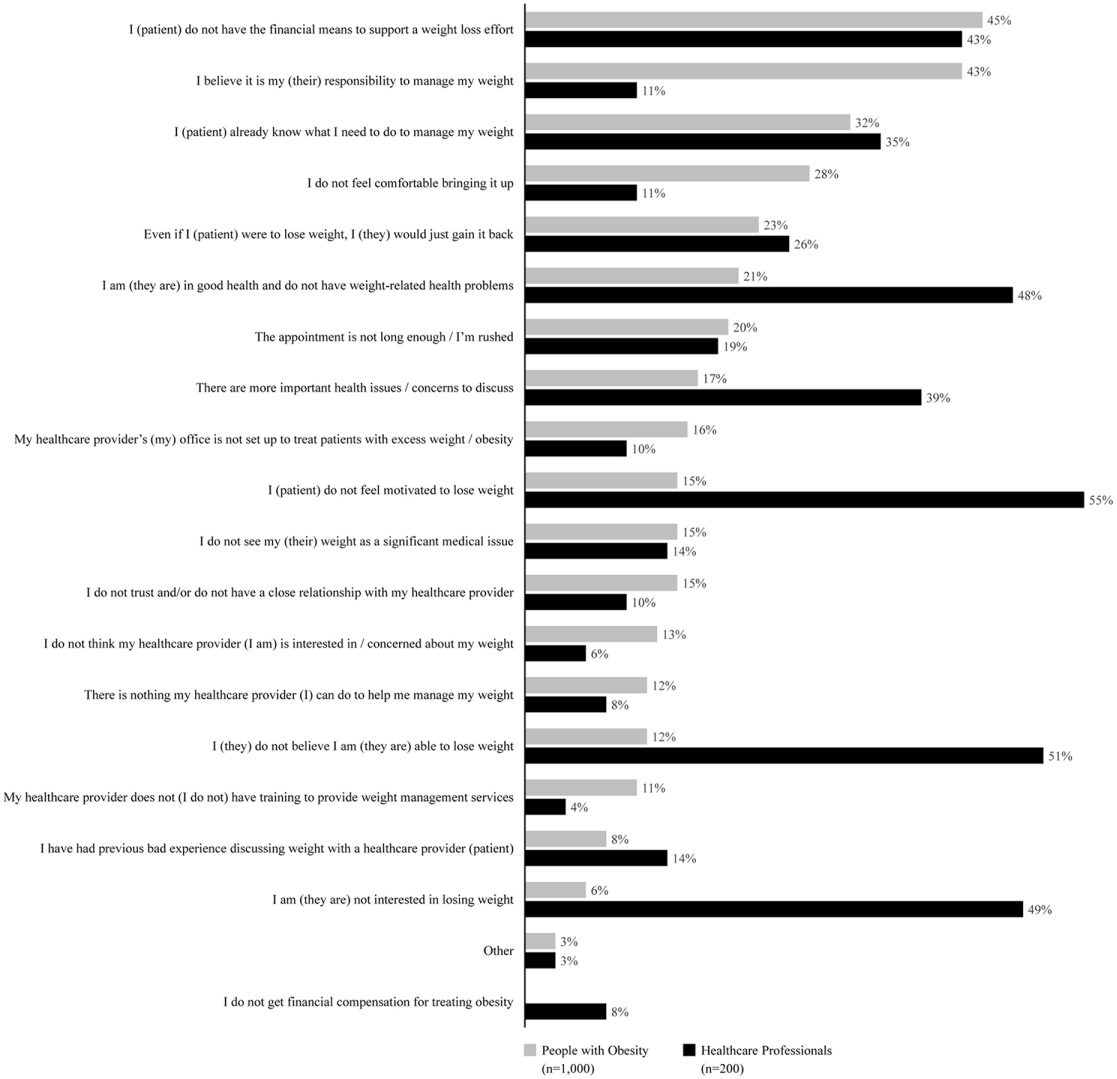
Reasons for not having weight discussion, as reported by people with obesity (PwO) and healthcare professionals (HCPs). Based upon the question to PwO, “Which of the following are/would be the top five reasons for which you might not discuss managing your weight with your healthcare provider?” and to HCPs, “What are the top 5 reasons for which you might not discuss obesity with a patient?”.

Nearly one in three PwO (30%) discussed weight with their HCPs in the past five years, while HCPs reported discussing weight with 44% of their PwO. When discussed, just over half (53%) of HCPs said they initiated the conversation while 61% PwO stated they initiated the weight conversation themselves. The main reasons cited by PwO for not discussing weight with HCPs were lack of financial means to support their WL efforts (45%), assuming self-responsibility for WL (43%) and the belief that they already know what they need to do to manage their weight (32%). HCPs considered PwOs lack of motivation (55%), disbelief in their ability to lose weight (51%) and PwO lack of interest (49%) as the top reasons for them not to initiate a WL discussion (Fig 3).

Although, most HCPs (87%) reported recording the obesity diagnosis in their patients’ charts most or all of the time, they informed only 52% of PwO of their diagnosis and scheduled follow-up appointments in only 43% of cases. The majority of PwO who discussed weight with their HCP in the past five years were most likely to visit a primary care physician (58%), followed by obesity specialist (23%), other HCPs (23%) and dieticians (22%).

### Sources of information used for managing weight

PwO reported relying on the internet for information on weight management, with 74% using social media, 50% using smartphone applications and only 33% consulting a HCP.

### Perceptions and attitudes towards prescription weight loss methods

On average, PwO set a WL goal of 20·1% of their total body weight. The most commonly recommended methods for weight management by HCPs included elimination diets (18%), specific diet or exercise programs (15%), and sleep quality management (15%) (S3 Fig).

Among PwO, only 31% viewed anti-obesity medications (AOMs) as a desirable option for weight management, and 46% reported they would prefer AOMs over bariatric surgery. In contrast, 20% of HCPs considered bariatric surgery as an effective option for weight management. Although 65% of HCPs believed their patients trusted them to prescribe WL medications, only 25% considered these medications to be the most helpful tool for weight management. Additionally, 22% of HCPs reported feeling uncomfortable prescribing WL medications, citing a lack of knowledge as a barrier.

**Fig 4:**
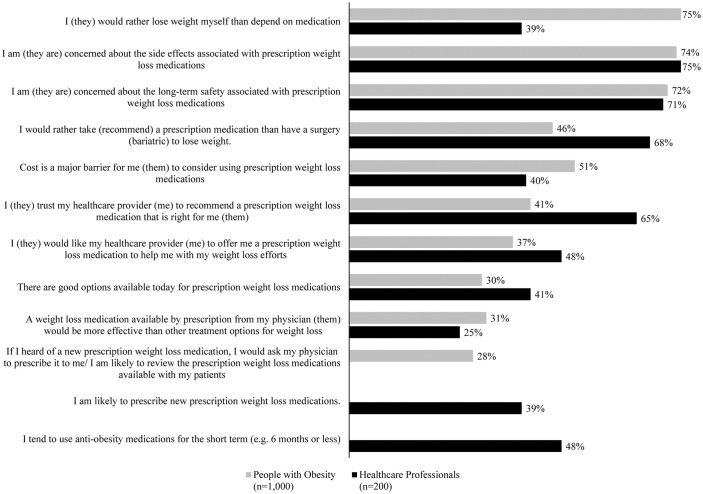
Attitudes toward prescription weight loss methods as reported by people with obesity (PwO) and healthcare professionals (HCPs). Percentage of PwO and HCPs who indicated their level of agreement was a 5 on a 5-point scale where 1 meant “Do not agree at all” and 5 meant “Completely agree.”.

Most PwO (75%) preferred losing weight independently rather than using AOMs. Nearly one in three PwO (30%) and 41% of HCPs acknowledged the availability of effective WL medications (Fig 4). However, both PwO and HCPs expressed concerns about the adverse effects (74% and 75%, respectively) and long-term safety (72% and 71%, respectively) of AOMs. Additionally, 51% of PwO considered cost as one of the primary barriers to considering prescription WL medications (Fig 4). Both PwO (82%) and HCPs (71%) preferred lifestyle changes over bariatric surgery for WL, viewing surgery as a last resort (41% of PwO and 58% of HCPs). While most PwO (58%) believed cost to be a major barrier to surgery, HCPs were less likely to view cost as a significant impediment (38%).

## Discussion

This cross-sectional, observational study assessed the perceptions, attitudes, behaviours and barriers towards obesity among PwO and HCPs across nine countries in the APAC region, including Indonesia. This manuscript aims to explore the alignment and differences between PwO and HCPs regarding their perceptions of obesity in Indonesia.

We found a striking discordance in agreement with the statement that obesity is a chronic disease between PwO (54%) and HCPs (90%).This misalignment is consistent with findings from other ACTION studies, such as ACTION APAC (68% and 84%) and other International ACTION studies: ACTION IO (68% and 88%), ACTION US (65% and 80%) and ACTION Canada (60% and 94% HCP) [6,8–10]. Recognizing the chronic nature of the disease is essential, as it warrants a long-term treatment approach; a lack of awareness may have an adverse influence on obesity management methods and eventually on achieving successful outcomes [11]. Not surprisingly, PwO showed a preference for lifestyle changes (diet and exercise) over AOMs or bariatric surgery for weight management. However, it is important to note that even among HCPs who acknowledged obesity as a chronic disease, lifestyle changes were the most commonly recommended option as opposed to using AOMs or bariatric surgery, even for PwO who are eligible for these interventions. Both were concerned about the long-term safety of AOMs, which may be attributed to the fact that at the time of the survey, the AOMs available in Indonesia were orlistat and diethylpropion (DEP). Interestingly, nearly 20% of HCPs reported that they are not comfortable prescribing AOMs due to a lack of knowledge. This highlights a need for additional training and increased awareness about the AOMs which could be accomplished by providing training materials or resources to support HCPs.

Furthermore, the lower acceptance of obesity as a chronic disease by PwO may be due to their limited understanding of the disease and its complex pathology, which might influence their attitudes towards obesity and its management. For example, PwO considered WL as solely their responsibility; they relied more on the internet for information related to obesity rather than consulting an HCP, and attributed weight regain to personal failures rather than on disease biology. Conversely, because HCPs had a better understanding of the disease, they put the onus on themselves to support PwO in WL efforts rather than leaving it to PwO discretion. However, HCPs expressed reservations about prescribing AOMs due to concerns about their adverse effects and long-term safety. Earlier reports highlighted similar attitudes among HCPs, attributing their reservations in prescribing AOMs to a lack of essential knowledge for effective utilization [12].

Notably, 67% of PwO perceived themselves as overweight or of normal weight despite meeting the criteria for obesity (BMI ≥ 25 kg/m^2^). These perceptions could be attributed to significant discrepancies in BMI cut-off values being used in defining obesity among Indonesian researchers. It is imperative to establish a standardized cut-off point and adopt a unified perspective in BMI measurement to enhance the accuracy of obesity diagnosis and the efficacy of subsequent clinical applications and research within the Indonesian population [5]. This necessitates a revision of the current obesity diagnostic criteria, and the formulation of standardized clinical guidelines tailored to the Indonesian population. Existing literature indicates that varying definitions and cutoffs for overweight/obesity diagnosis in Indonesia preclude consistent comparisons of prevalence rates among most studies [5,13].

Furthermore, fewer (30%) PwO in Indonesia reported discussing weight with their HCP compared to those in ACTION APAC (43%), ACTION US (71%) and ACTION IO (54%) and ACTION Canada studies (54%) [6, 8 − 10]. The reasons provided by PwO for not discussing their weight included a lack of financial means to support WL efforts, the belief that managing weight is their responsibility, and a feeling that they already know what needs to be done. The lack of reimbursement for obesity-related treatments in Indonesia could further compound these barriers. Additionally, most PwO seeking treatment for obesity in Indonesia are from the middle socio-economic class, with the majority earning less than 3,070,000 Indonesian Rupiah (IDR) monthly (approximately 195 USD). As a result, they may not consider obesity a disease and may not prefer to allocate a budget for its treatment. It could also be argued that not considering obesity as a chronic disease and unawareness about its long-term implications on their health might also have contributed to PwO’s preference. Also, cultural differences between Asians and their Western counterparts play an important role. Asian individuals are more likely to self-blame for their weight, worry about societal perceptions, and perceive their weight as either normal or overweight, assuming self-responsibility. Additionally, PwO often prefer self-directed WL strategies and believe that lifestyle changes alone are sufficient. These behaviors could collectively contribute to fewer PwO discussing their weight with HCPs. Similar barriers have been reported in the literature as common reasons for PwO not initiating conversations about weight management [14,15].

Overall, the aforementioned misperceptions, might have led to an average wait of three years before PwO discusses their weight with an HCP. Additionally, the disparity between PwO’s self-reported WL efforts and HCPs’ observations (44% vs. 50%), along with the high incidence of weight regain (68%) after initial success, highlights the complexities of maintaining long-term weight control. In this survey, 70% PwO indicated that they are motivated to lose weight and 50% of HCPs agreed to it. However, this motivation level among PwO did not translate into actions towards weight management. For example, PwO set themselves a goal of losing 20% of their body weight but relied on only diet and exercise, which may not result in losing >5–10% of their body weight. Furthermore, PwO were hesitant to seek guidance from HCP and relied on online resources for weight-related information. Compounding these perceptions, most PwO considered themselves to be living with either normal or overweight despite meeting the criteria for obesity (as per the inclusion criteria).

Among PwO who discussed their weight with an HCP, nearly half (48%) reported not receiving a formal diagnosis of obesity. This could be influenced by the consulting HCP’s speciality. Only 23% of PwO visited an obesity specialist, possibly due to a lack of awareness of this specialty in Indonesia. Furthermore, weight stigma is widespread among PwO in Indonesia. It is pervasive and can negatively influence clinical decisions, and public health communications [16–18].

The survey findings highlight the need for more affordable obesity care options in Indonesia. At the same time, current obesity treatment guidelines advocate for a multidisciplinary approach for weight management, encompassing lifestyle changes, behavioural therapy, medication, and/or bariatric surgery when appropriate [19,20].

This is the first study focusing on the perceptions and attitudes on obesity and its management in Indonesia involving a large population of PwO and HCPs, with the latter representing both primary care and specialists. The survey also included aspects of weight bias and stigma, as they influence attitudes and perceptions of PwO. We believe that this is a strength of the study, along with the anonymous, self-reported design, which may have encouraged people to be more honest in their responses.

Limitations of this study are consistent with those reported in the broader ACTION APAC study [6], including the cross-sectional survey design [21], reliance on self-reported height and weight, potentially leading to an underestimation of BMI [22], and the accuracy of respondent recall bias [23]. Additionally, the survey responses may not fully represent the opinions of all PwO and HCPs in the region, potentially impacting the generalizibility of the results. However, the limitations in the current study are a common challenge in survey methodologies and should be considered when interpreting the findings.

## Conclusion

Our findings suggest that PwO were less likely to recognize obesity as a chronic disease. Most perceived themselves to be living either with normal or overweight and assumed self-responsibility to lose weight and relied on online resources for weight-related information. While most HCPs acknowledged obesity as a chronic disease, they recommended lifestyle changes over AOMs as obesity management methods often citing concerns about the long-term safety of AOMs. Overall, this survey highlighted gaps in understanding and management of obesity among both PwO and HCPs in Indonesia emphasizing a need for increased disease awareness. The findings suggest that improving HCP training through a standardised obesity curriculum, developing country-specific guidelines, providing accessible educational resources for PwO, and ensuring the availability of AOMs are essential to overcome barriers and support effective obesity management in Indonesia.

A comprehensive approach to obesity care is crucial in ensuring that both PwO and HCPs in Indonesia are equipped with the knowledge and resources necessary to navigate the complexities of this chronic disease, ultimately leading to improved health outcomes and quality of life for PwO.

## Supporting information

S1 Fig Motivators for weight loss as reported by people with obesity (PwO) and healthcare professionals (HCPs). Percentage of PwO and HCPs selecting each response.
(TIF)

S2 Fig Perceived barriers to weight loss among people with obesity (PwO) and Healthcare professionals (HCPs). Percentage of PwO and HCPs who indicated their level of agreement was a 5 on a 5-point scale where 1 meant “Do not agree at all” and 5 meant “Completely agree.”.
(TIF)

S3 Fig Weight management methods recommended by healthcare professionals. (TIF)

S1 File People with Obesity (PwO) Questionnaire. (DOCX)

S2 File Healthcare Professional (HCP) Questionnaire. (DOCX)

S3 File Translation check and validation of survey. (DOCX)
