# Supplementary material for: Perceptions, attitudes, behaviours and barriers towards obesity among people with obesity and health care professionals in Indonesia: An exploratory online survey
Source: PLoS One. 2026 Jun 4;21(6):e0350857. doi: 10.1371/journal.pone.0350857 (PMC13235876; doi:10.1371/journal.pone.0350857)
Supplement: S1 File — (DOCX) [file pone.0350857.s004.docx]

­KJT Group, Inc.

6 East St

Honeoye Falls, NY, 14472

**Awareness, Care & Treatment In Obesity MaNagement -**

**ACTION APAC - PwO Questionnaire**

**KJT Group Contacts:**

| Lynn Clement  Email: lynn@kjtgroup.com  Phone: 585-624-8050 x304 | Andrea Stoltz  Email: andreas@kjtgroup.com  Phone: 585-624-8050 x333 |  |  |
| --- | --- | --- | --- |
| Nick Henderson  Email: nickh@kjtgroup.com  Phone: 585-624-8050 x348  Rebecca Hahn  Email: rebeccah@kjtgroup.com  Phone: 585-624-8050 x312 | Peg Jaynes  Email: pegj@kjtgroup.com  Phone: 585-624-8050 x353 |  |  |

**Recruitment:**

|  | **Indonesia** | **India** | **Pakistan** | **Thailand** | **Malaysia** | **Singapore** | **Philippines** | **Vietnam** | **Bangladesh** | **TOTAL** |  |
| --- | --- | --- | --- | --- | --- | --- | --- | --- | --- | --- | --- |
| **PwO** | 1000 | 2000 | 1000 | 1500 | 1000 | 1000 | 1000 | 1000 | 1000 | 10,500 |  |

**Screening Criteria:**

- Age 18+
- Current BMI 25+ calculated based on self-reported height and weight (for all countries EXCEPT Singapore)
- Current BMI 27+ calculated based on self-reported height and weight (Singapore only)
- Not currently pregnant
- Does not participate in intense fitness or body building programs
- Targeting based on age, gender, education, and region

Respondents will be further classified according to the following:

- - **Maintaining weight loss**: Lost at least 10% of body weight in the past 3 years and has kept weight off for at

least a year

- - **Committed to Action**: Intend to take action to lose weight or committed to/enrolled in a plan to lose weight

Data Validation Questions:

**q435, q500_11, q640_8**

**SECTION S: Screener**

**ALL RESPONDENTS**

**S0.** Thank you for taking the time to participate in this important research. For your convenience we are offering this survey in multiple languages. Please select the language that you are most familiar with.

1. English [DISPLAY FOR ALL]
2. Bengali / Bangla [DISPLAY FOR BANGLADESH]
3. Hindi [DISPLAY FOR INDIA AND PAKISTAN]
4. Indonesian [DISPLAY FOR INDONESIA]
5. Bahasa Malaysia / Malay [DISPLAY FOR MALAYSIA]
6. Urdu [DISPLAY FOR PAKISTAN]
7. Tagalog / Filipino [DISPLAY PHILIPPINES]
8. Malay [DISPLAY FOR SINGAPORE]
9. Thai [DISPLAY FOR THAILAND]
10. Vietnamese [DISPLAY FOR VIETNAM]
11. Standard Chinese (Mandarin) [DISPLAY FOR SINGAPORE, MALAYSIA]
12. Tamil [DISPLAY FOR SINGAPORE, MALAYSIA]

**ALL RESPONDENTS**

**S1** Thank you for your interest in this survey on important healthcare issues!

Before starting, KJT Group requires you to read the following information:

- KJT Group is a **global market research company** who is asking you to participate in this research survey. KJT Group is the data controller.
- KJT’s legal basis for the processing of your personal data is your consent.
- Your participation is fully voluntary; you can choose to stop at any time and you can, after completion of the questionnaire, withdraw your consent at any time by writing to [help@kjtgroup.com](mailto:help@kjtgroup.com) without any penalty or loss of benefits to which you are otherwise entitled.
- The purpose of the survey is to help the sponsor (Novo Nordisk) understand the healthcare experiences of patients.
- The risk of the research is to your privacy. Your responses will be kept **completely confidential** and will never be connected with your name. Your alternative is to not participate in the survey.
- You have the **right to see and get a copy your data, amend your data, or erase your data** at any time.
- We expect, on average, it will take about **20 minutes** to complete this survey.
- Your responses will be aggregated with other responses in KJT Group’s research report and will be made publicly available in a peer-reviewed scientific journal publication once the study has finished. However, **your name will never be included in the report, publication, or identified to the sponsor**.
- Your responses will be transferred to and **stored on secure servers in the United States**.
- We will **destroy your personally identifiable information within 12 months of data collection**; however, we will maintain a permanent record of this consent.
- If you do not qualify for the study, your personal data will be stored electronically by KJT Group and erased no later than 12 months after the end of data collection.
- All information from this study will be stored for at least 5 years after the Study Report is made, or according to local requirements. The study report contains the full results of the study.
- Your personal information is protected by the data protection law as applicable in your country.
- Your **participation is voluntary,** and you may choose to stop participating at any time (withdraw consent) without any penalty or loss of benefits to which you are otherwise entitled.
- If you qualify and complete this survey, you will receive the online panel credit listed in your invitation. There are no costs to you for your participation.
- KJT Group will make sure that the information we ask about you cannot be looked at by people who are not authorized to do so. To make sure that the study is done correctly and to check the results, the following people will be able to see your study information:
  - The Research Ethics Committee/Institutional Review Board
  - National medicine authorities from other countries
- A description of this clinical trial will be available on http://www.ClinicalTrials.gov, as required by U.S. Law. This Web site will not include information that can identify you. At most, the Web site will include a summary of the results. You can search this Web site at any time.

You may contact 585-624-8050 (24 hours) or [help@kjtgroup.com](mailto:help@kjtgroup.com) with questions or concerns, or if you would like to follow-up on these points or need additional support.

• Protocol number: DAS-006

• IRB Protocol number: 20220349

• Investigator: Rebecca Hahn, MPH, 777 Canal View Blvd, Ste 1400 Rochester, New York 14623-2828 United States

This research is being overseen by WCG IRB. An IRB is a group of people who perform independent review of research studies. You may talk to them at 855-818-2289 or [researchquestions@wcgirb.com](mailto:researchquestions@wcgirb.com) if:

- You have questions, concerns, or complaints that are not being answered by the research team.
- You are not getting answers from the research team.
- You cannot reach the research team.
- You want to talk to someone else about the research.
- You have questions about your rights as a research subject.

Do you agree to these terms and want to continue with the survey?

1. Yes [CONTINUE]
2. No [TERMINATE]

**ALL RESPONDENTS**

**S2.** Thank you. This survey is focused on your personal experiences, and we very much appreciate your honesty in responding. To begin, we would like to gather some basic information to be used for categorization purposes.

In what year were you born?

*Please enter as a four-digit number, e.g., 1963.*

[RANGE: 1920-2020]

|_|_|_|_|

**ALL RESPONDENTS**

**S3** HIDDEN COMPUTE FOR AGE

[IF AGE 18 OR OLDER (S3>17) ASK S4. ELSE TERMINATE]

**AGE 18 OR OLDER (S3>17)**

**S4.** Which gender do you identify with?

1. Male
2. Female
3. Other [DO NOT DISPLAY FOR INDONESIA, PAKISTAN OR MALAYSIA]

**AGE 18 OR OLDER (S3>17)**

**S5.** REGION QUESTION CUSTOM FOR EACH COUNTRY

[FOR BANGLADESH DISPLAY: In which division is your primary residence located?

[INSERT DROP DOWN MENU; ALPHA SORT]

1. Barishal
2. Chittagong
3. Dhak or Mymensingh
4. Khulna
5. Rajshaha
6. Rangpur
7. Sylhet

99. I do not live in Bangladesh [ANCHOR] [TERMINATE]

[IF LIVES IN BANGLADESH (S5/NE99) CONTINUE. ELSE TERMINATE]

[FOR INDIA DISPLAY:

In which state or territory is your primary residence located?

[INSERT DROP DOWN MENU; ALPHA SORT]

States:

1. Andhra Pradesh
2. Arunachal Pradesh
3. Assam
4. Bihar
5. Chhattisgarh
6. Goa
7. Gujarat
8. Haryana
9. Himachal Pradesh
10. Jharkhand
11. Karnataka
12. Kerala
13. Madhya Pradesh
14. Maharashtra
15. Manipur
16. Meghalaya
17. Mizoram
18. Nagaland
19. Odisha
20. Punjab
21. Rajasthan
22. Sikkim
23. Tamil Nadu
24. Telangana
25. Tripura
26. Uttar Pradesh
27. Uttarakhand
28. West Bengal

Union Territories:

1. Andaman and Nicobar Islands
2. Chandigarh
3. Dadra & Nagar Haveli and Daman & Diu
4. Delhi
5. Jammu and Kashmir
6. Lakshadweep
7. Puducherry
8. Ladakh

99. I do not live in India [ANCHOR] [TERMINATE]

[IF LIVES IN INDIA (S5/NE99) CONTINUE. ELSE TERMINATE]

HIDDEN VARIABLE:

S5A [REGIONAL RECODES INDIA]

1. Andhra Pradesh [S5=1]
2. Bihar [S5=4]
3. Gujarat [S5=7]
4. Karnataka [S5=11]
5. Madhya Pradesh [S5=13]
6. Maharashtra [S5=14]
7. Rajasthan [S5=21]
8. Tamil Nadu [S5=23]
9. Uttar Pradesh [S5=26]
10. West Bengal [S5=28]
11. All other regions [S5=2,3,5,6,8,9,10,12,15,16,17,18,19,20,22,24,25,27,29-36]

[FOR INDONESIA DISPLAY:

In which region is your primary residence located?

[INSERT DROP DOWN MENU; ALPHA SORT]

1. Jabodetabek
2. West Java
3. Banten
4. Central Java
5. Yogyakarta
6. East Java
7. Sumatera
8. Sulawesi
9. Kalimantan
10. Bali-Nusa Tenggara
11. Maluku-Papua

99. I do not live in Indonesia [ANCHOR] [TERMINATE]

[IF LIVES IN INDONESIA (S5/NE99) CONTINUE. ELSE TERMINATE]

[HIDDEN VARIABLE:

S5A [REGIONAL RECODE FOR INDONESIA]

1. Java [S5=1-6]
2. Sumatera [S5=7]
3. Sulawesi [S5=8]
4. Kalimantan [S5=9]
5. Bali-Nusa Tenggara [S5=10]
6. Maluku-Papua [S5=11]

[FOR MALAYSIA DISPLAY:

In which region is your primary residence located?

[INSERT DROP DOWN MENU; ALPHA SORT]

1. Selangor
2. Johor
3. Sabah
4. Sarawak
5. Perak
6. Kedah
7. W.P. Kuala Lumpur
8. Pulau Pinang
9. Kelatan
10. Pahang
11. Terengganu
12. Negeri Sembilan
13. Melaka
14. Perlis
15. W.P. Labuan
16. W.P. Putrajaya

99. I do not live in Malaysia [ANCHOR] [TERMINATE]

[IF LIVES IN MALAYSIA (S5/NE99) CONTINUE. ELSE TERMINATE]

[HIDDEN VARIABLE:

S5A [REGIONAL RECODES MALAYSIA]

1. Selangor [S5=1]
2. Johor [S5=2]
3. Sabah [S5=3]
4. Sarawak [S5=4]
5. Perak [S5=5]
6. Kedah [S5=6]
7. W.P. Kuala Lumpur [S5=7]
8. Pulau Pinang [S5=8]
9. Kelatan [S5=9]
10. Pahang [S5=10]
11. All other regions [S5=11]

[FOR PAKISTAN DISPLAY:

In which province or territory is your primary residence located?

[INSERT DROP DOWN MENU; ALPHA SORT]

1. Balochistan
2. Punjab
3. Sindh
4. Khyber Pakhtunkhwa
5. Islamabad
6. Gilgit Baltistan
7. Azad Jammu and Kashmir

99. I do not live in Pakistan [ANCHOR] [TERMINATE]

[IF LIVES IN PAKISTAN (S5/NE99) CONTINUE. ELSE TERMINATE]

[FOR PHILIPPINES DISPLAY:

In which region is your primary residence located?

[INSERT DROP DOWN MENU; ALPHA SORT]

1. Calabarzon
2. National Capital Region
3. Central Luzon Region
4. Central Visayas
5. Western Visayas
6. Bicol
7. Ilocos Region
8. Davao Region
9. Northern Mindanao
10. Soccsksargen
11. Eastern Visayas
12. Bangsamoro Autonomous Region in Muslim Mindanao (BARMM)
13. Zamboanga Peninsula
14. Cagayan Valley Region
15. Mimaropa Region
16. Caraga
17. Cordillera (CAR)

99. I do not live in Philippines [ANCHOR] [TERMINATE]

[IF LIVES IN PHILIPPINES (S5/NE99) CONTINUE. ELSE TERMINATE]

[HIDDEN VARIABLE:

S5A [REGIONAL RECODES PHILIPPINES]

1. Calabarzon [S5=1]
2. National Capital [S5=2]
3. Central Luzon [S5=3]
4. Central Visayas [S5=4]
5. Western Visayas [S5=5]
6. Bicol [S5=6]
7. Ilocos [S5=7]
8. Davao [S5=8]
9. Northern Mindanao [S5=9]
10. Soccsksargen [S5=10]
11. All other regions [S5=11-17]

[FOR SINGAPORE DISPLAY:

In which region is your primary residence located?

[INSERT DROP DOWN MENU; ALPHA SORT]

1. North-East
2. Central
3. West
4. East
5. North

99. I do not live in Singapore [ANCHOR] [TERMINATE]

[IF LIVES IN SINGAPORE (S5/NE99) CONTINUE. ELSE TERMINATE]

[FOR THAILAND DISPLAY:

In which region is your primary residence located?

[INSERT DROP DOWN MENU; ALPHA SORT]

1. Bangkok
2. Central Region (excluding Bangkok)
3. Northern Region
4. Northeastern Region
5. Southern Region

99. I do not live in Thailand [ANCHOR] [TERMINATE]

[IF LIVES IN THAILAND (S5/NE99) CONTINUE. ELSE TERMINATE]

[FOR VIETNAM DISPLAY:

In which region is your primary residence located?

[INSERT DROP DOWN MENU; ALPHA SORT]

1. Northern Midlands and Mountains
2. Red River Delta
3. North and South Central Coast
4. Central Highlands
5. Southeast
6. Mekong River Delta

99. I do not live in Vietnam [ANCHOR] [TERMINATE]

[IF LIVES IN VIETNAM (S5/NE99) CONTINUE. ELSE TERMINATE]

**LIVES IN MALAYSIA (Malaysia S5/1-16)**

**S20_Mal** Which of the following best describes your ethnicity?

1. Malay
2. Chinese
3. Malay Indian
4. Other ethnicity

**AGE 18 OR OLDER (S3>17)**

**S6.** [DISPLAY FOR BANGLADESH, INDIA, PAKISTAN, VIETNAM: In 2021, what was your household’s total **yearly** income before taxes?]

[DISPLAY FOR INDONESIA, MALAYSIA, PHILIPPINES, SINGAPORE, THAILAND: In 2021, what was your household’s total **monthly** income before taxes?]

*Please remember that your individual information will never be shared. These questions are only used to ensure a representative mix of respondents is achieved.*

[FOR BANGLADESH DISPLAY:

[INSERT DROP DOWN MENU]

1. $1,025 or less
2. $1,026 to $4,035
3. $4,036 to $12,475
4. $12,476 or more
5. Decline to answer

[FOR INDIA DISPLAY:

[INSERT DROP DOWN MENU]

1. Less than 25,000 rupees
2. 25,000 to 49,999 rupees
3. 50,000 to 99,999 rupees
4. 100,000 to 199,999 rupees
5. 200,000 to 399,999 rupees
6. 400,000 or more
7. Decline to answer

]

[FOR INDONESIA DISPLAY:

[INSERT DROP DOWN MENU]

1. Less than 3,070,000 IDR
2. 3,070,000 to 5,519,999 IDR
3. 5,520,000 to 11,399,999 IDR
4. 11,400,000 to 30,799,999 IDR
5. 30,800,000 or more
6. Decline to answer

[FOR MALAYSIA DISPLAY:

[INSERT DROP DOWN MENU]

1. Less than RM 2,500
2. RM 2,501 to RM 3,169
3. RM 3,170 to RM 3,969
4. RM 3,970 to RM 4,849
5. RM 4,850 to RM 5,879
6. RM 5,880 to RM 7,099
7. RM 7,100 to RM 8,699
8. RM 8,700 to RM 10,959
9. RM 10,960 to RM 15,039
10. RM 15,040 or more
11. Decline to answer

[FOR PAKISTAN DISPLAY:

[INSERT DROP DOWN MENU]

1. Lower income
2. Lower middle income
3. Middle income
4. Upper middle income
5. Upper income
6. Decline to answer

[FOR PHILIPPINES DISPLAY:[INSERT DROP DOWN MENU]

1. Below P10,957
2. P10,957 to P21,914
3. P21,915 to P43,828
4. P43,829 to P76,668
5. P76,669 to P131,484
6. P131,485 or more
7. Decline to answer

[FOR SINGAPORE DISPLAY:

[INSERT DROP DOWN MENU]

1. Less than $1,000 SGD
2. $1,000 - $2,499 SGD
3. $2,500 - $4,999 SGD
4. $5,000 - $7,499 SGD
5. $7,500 - $9,999 SGD
6. $10,000 - $14,999 SGD
7. $15,000 - $19,999 SGD
8. $20,000 or more
9. Decline to answer

[FOR THAILAND DISPLAY:

[INSERT DROP DOWN MENU]

1. <1,500 Baht
2. 1,500-3,000 Baht
3. 3,001-5,000 Baht
4. 5,001-10,000 Baht
5. 10,001-15,000 Baht
6. 15,001-30,000 Baht
7. 30,001-50,000 Baht
8. 50,001-100,000 Baht
9. 100000+ Baht
10. Decline to answer

[FOR VIETNAM DISPLAY:

[INSERT DROP DOWN MENU]

1 No income

2) 1-9,999 VND

3) 10,000 - 19,999 VND

4) 20,000 - 29,999 VND

5) 30,000 - 39,999 VND

6) 40,000 - 49,999 VND

7) 50,000 - 59,999 VND

8) 60,000 or more VND

9) Decline to answer

**AGE 18 OR OLDER (S3>17)**

**S7.** What is the highest level of education you have completed, or the highest degree you have received?

[FOR BANGLADESH DISPLAY:

[INSERT DROP DOWN MENU]

1. No education
2. Lower secondary education
3. Upper secondary education
4. Bachelor’s degree
5. Post-graduate (master’s degree or above)

[FOR INDIA DISPLAY:

[INSERT DROP DOWN MENU]

1. Less than Primary
2. Primary
3. Middle
4. Matric/Secondary
5. Higher Secondary/Intermediate
6. Graduate or above
7. Diploma, Certificate, Other

[FOR INDONESIA DISPLAY:

[INSERT DROP DOWN MENU]

1. Below upper secondary education
2. Upper secondary or post-secondary, non-tertiary
3. Tertiary

[FOR MALAYSIA DISPLAY:

[INSERT DROP DOWN MENU]

1. Primary level
2. Lower Secondary level
3. Upper Secondary level or higher

FOR PAKISTAN DISPLAY:

[INSERT DROP DOWN MENU]

1. Below primary
2. Primary
3. Middle
4. Matric
5. Intermediate
6. Graduate
7. Masters and above
8. Diploma/certificate
9. Other

FOR PHILIPPINES DISPLAY:

[INSERT DROP DOWN MENU]

1. No Grade Completed
2. Elementary Undergraduate
3. Elementary Graduate
4. Junior High School Undergraduate
5. Junior High School Completed
6. Senior High School Undergraduate
7. Senior High School Graduate
8. Post-Secondary Undergraduate
9. Post-Secondary Graduate
10. College Undergraduate
11. College Graduate

FOR SINGAPORE DISPLAY:

[INSERT DROP DOWN MENU]

1. Pre-Primary
2. Primary
3. Secondary
4. Post-Secondary (Non-Tertiary)
5. Polytechnic Diploma
6. Professional Qualification and Other Diploma
7. University

FOR THAILAND DISPLAY:

[INSERT DROP DOWN MENU]

1. No education
2. Elementary level
3. Secondary level
4. Higher education (diploma, undergraduate, masters, doctorate)
5. Religious or Unknown

FOR VIETNAM DISPLAY:

[INSERT DROP DOWN MENU]

1. Under Primary
2. Primary
3. Lower Secondary
4. Upper Secondary
5. Pre-Intermediate
6. Intermediate
7. College
8. University
9. Masters or Doctorate

**S8. DEMOGRAPHIC TARGETS (%)**

| Category | Targets by Country | | | | | | | | |
| --- | --- | --- | --- | --- | --- | --- | --- | --- | --- |
|  | **Bangladesh** | **India** | **Indonesia** | **Malaysia** | **Pakistan** | **Philippines** | **Singapore** | **Thailand** | **Vietnam** |
| Region |  |  |  |  |  |  |  |  |  |
| Region code 1 | 6% | 7% | 56% | 19% | 6% | 15% | 23% | 10% | 19% |
| Region code 2 | 20% | 9% | 22% | 12% | 53% | 12% | 23% | 23% | 34% |
| Region code 3 | 33% | 5% | 7% | 11% | 23% | 11% | 23% | 19% | 30% |
| Region code 4 | 11% | 5% | 6% | 9% | 15% | 7% | 17% | 34% | 9% |
| Region code 5 | 13% | 6% | 6% | 8% | 1% | 7% | 14% | 13% | 27% |
| Region code 6 | 11% | 9% | 3% | 7% | 1% | 6% |  |  | 26% |
| Region code 7 | 7% | 6% |  | 6% | 1% | 5% |  |  |  |
| Region code 8 |  | 6% |  | 6% |  | 5% |  |  |  |
| Region code 9 |  | 17% |  | 5% |  | 5% |  |  |  |
| Region code 10 |  | 8% |  | 5% |  | 5% |  |  |  |
| Region code 11 |  | 22% |  | 12% |  | 22% |  |  |  |
| Gender |  |  |  |  |  |  |  |  |  |
| Male | 50% | 51% | 51% | 51% | 51% | 50% | 49% | 49% | 50% |
| Female | 50% | 49% | 49% | 49% | 49% | 50% | 51% | 51% | 50% |
| Other | <1% | <1% | NA | NA | NA | <1% | <1% | <1% | <1% |
| Age |  |  |  |  |  |  |  |  |  |
| 18-24 years | 22% | 22% | 16% | 18% | 22% | 20% | 10% | 15% | 13% |
| 25-34 years | 21% | 21% | 20% | 23% | 28% | 24% | 19% | 25% | 24% |
| 35-44 years | 22% | 19% | 20% | 20% | 19% | 20% | 20% | 23% | 20% |
| 45-54 years | 21% | 18% | 17% | 14% | 14% | 15% | 19% | 15% | 18% |
| 55-64 years | 7% | 12% | 15% | 13% | 11% | 11% | 18% | 12% | 13% |
| 65 and over | 7% | 8% | 12% | 12% | 6% | 10% | 14% | 10% | 12% |
| Education |  |  |  |  |  |  |  |  |  |
| Education code 1 | 8% | 27% | 59% | 6% | 13% | 4% | 8% | 8% | 10% |
| Education code 2 | 34% | 28% | 28% | 33% | 27% | 21% | 34% | 59% | 21% |
| Education code 3 | 19% | 19% | 13% | 61% | 21% | 11% | 24% | 24% | 32% |
| Education code 4 | 31% | 12% |  |  | 19% | 16% | 8% | 8% | 17% |
| Education code 5 | 8% | 8% |  |  | 9% | 21% | 10% | 1% | 3% |
| Education code 6 |  | 5% |  |  | 6% | 2% | 2% |  | 4% |
| Education code 7 |  | 1% |  |  | 3% | 2% | 14% |  | 3% |
| Education code 8 |  |  |  |  | 1% | 1% |  |  | 9% |
| Education code 9 |  |  |  |  | 1% | 2% |  |  | 1% |
| Education code 10 |  |  |  |  |  | 8% |  |  |  |
| Education code 11 |  |  |  |  |  | 12% |  |  |  |

[IF FEMALE OR OTHER (S4/2-3) ASK S9. ELSE CONITNUE]

**FEMALE OR OTHER (S4/2-3)**

**S9.** Are you currently pregnant?

1. Yes TERMINATE
2. No CONTINUE

**AGE 18 OR OLDER (S3>17)**

**S10.** Do you participate in intense fitness or body building programs and consider yourself extremely fit?

1. Yes TERMINATE
2. No CONTINUE

**AGE 18 OR OLDER (S3>17)**

**S11.** What is your height?

*Your best estimate will do.*

*Please enter your height in centimeters.*

[RANGE 75-250]

Centimeters: |_|_|_|

**AGE 18 OR OLDER (S3>17)**

**S12.** What is your current weight?

*Please be as exact as possible.*

[RANGE 1-999]

Kilograms: |_|_|_|

**S13.** HIDDEN QUESTION FOR BMI CALCULATION

S11 convert to m, where 1 m = 100 cm

BMI = S12 kg / [S11 m ^2]

*Example: S12 Weight = 68 kg, S11 Height = 165 cm (1.65 m)*

*Calculation: 68 ÷ (1.65)2 = 24.98*

**S14.** HIDDEN QUESTION FOR WEIGHT CLASSIFICATION

[FOR ALL COUNTRIES EXCEPT SINGAPORE DISPLAY:

1. Not overweight (BMI < 25) |_|_|_| %
2. Obesity class 1 (BMI 25 to 29.9) |_|_|_| %
3. Obesity class 2 (BMI 30 to 34.9) |_|_|_| %
4. Obesity class 3 (BMI 35 to 39.9) |_|_|_| %
5. Obesity class 4 (BMI 40+) |_|_|_| %

[FOR SINGAPORE DISPLAY:

1. Not overweight (BMI < 27) |_|_|_| %
2. Obesity class 1 (BMI 27 to 31.9) |_|_|_| %
3. Obesity class 2 (BMI 32 to 36.9) |_|_|_| %
4. Obesity class 3 (BMI 37 to 41.9) |_|_|_| %
5. Obesity class 4 (BMI 42+) |_|_|_| %

[for all countries except singapore, IF CURRENT BMI 25+ (S13 ≥ 25) ASK S15. ELSE, TERMINATE.]

[FOR SINGAPORE IF CURRENT BMI 27+ (S13 ≥ 27) ASK S15. ELSE, TERMINATE]

**BMI 25+ (S13 > 25) FOR ALL COUNTRIES EXCEPT SINGAPORE, BMI 27+ (S13 > 27) FOR SINGAPORE**

**S15.** What is the **least** you have weighed in the past 3 years?

*Your best estimate will do.*

[RANGE 0 - S12-1]

Kilograms: |_|_|_|

99. [ ] I currently weigh the least I’ve weighed in the past 3 years [EXCLUSIVE]

[IF CURRENTLY MIN WEIGHT (S15/99) AUTO FILL S15 WITH S12]

**BMI 25+ (S13 > 25) FOR ALL COUNTRIES EXCEPT SINGAPORE, BMI 27+ (S13 > 27) FOR SINGAPORE**

**S16.** What is the **most** you have weighed in the past 3 years?

*[IF FEMALE OR OTHER (S4/2-3) show: “Please do not consider pregnancy.”]*

*Your best estimate will do.*

[RANGE S12 +1 - 999]

Kilograms: |_|_|_|

99. [ ] I currently weigh the most I’ve weighed in the past 3 years [EXCLUSIVE]

[IF CURRENTLY MAX WEIGHT (S16/99) AUTO FILL S16 WITH S12]

**BMI 25+ (S13 > 25) FOR ALL COUNTRIES EXCEPT SINGAPORE, BMI 27+ (S13 > 27) FOR SINGAPORE**

**S17.** In the past 6 months have you had significant weight loss due to major injury or illness (e.g., cancer, accident)?

1. Yes [TERMINATE]
2. No [CONTINUE]

**BMI 25+ (S13 > 25) FOR ALL COUNTRIES EXCEPT SINGAPORE, BMI 27+ (S13 > 27) FOR SINGAPORE**

**S18.** HIDDEN QUESTION FOR PERCENT WEIGHT LOSS

PERCENT WEIGHT LOSS = ((S16 - S12) / S16)*100

[IF AT LEAST 5% WEIGHT LOSS (S18≥5) ASK S19. ELSE JUMP TO S22]

**AT LEAST 5% WEIGHT LOSS (S18≥5)**

**S19.** You indicated your current weight is less than your maximum weight within the past 3 years. For how long would you say you’ve been able to maintain your weight loss?

1. 6 months or less
2. More than 6 months but less than a year
3. A year or more
4. I have not been able to maintain weight loss

**AT LEAST 5% WEIGHT LOSS (S18≥5)**

**S20.** You indicated your current weight is less than your maximum weight within the past 3 years. How successful do you feel you have been in losing weight?

1. Not at all successful
2. Not very successful
3. Somewhat successful
4. Very successful
5. Extremely successful

**BMI 25+ (S13 > 25) FOR ALL COUNTRIES EXCEPT SINGAPORE, BMI 27+ (S13 > 27) FOR SINGAPORE**

**S21.** HIDDEN QUESTION FOR WEIGHT LOSS STATUS

1. MAINTAINING WEIGHT LOSS (GET IF 10%+ LOSS AND MORE THAN 1 YEAR SUCCESS AND NOT ILLNESS/INJURY (S18 >= 10 AND S19/3)
2. NOT CURRENTLY MAINTAINING WEIGHT LOSS (GET IF LESS THAN 10% LOSS OR NOT MORE THAN 1 YEAR SUCCESS (S18 < 10 OR S19/1,2,4)

**BMI 25+ (S13 > 25) FOR ALL COUNTRIES EXCEPT SINGAPORE, BMI 27+ (S13 > 27) FOR SINGAPORE**

**S22.** Which of the following statements best describes you today?

1. I am **not** concerned about my weight, and I **have no plans** for weight loss within the next 6 months
2. I am concerned about my weight, but I **have no plans** for weight loss within the next 6 months
3. I am aware I have excess weight, and am **seriously considering** taking action to lose weight
4. I am aware I have excess weight, and I intend to take action to lose weight **within the next month**
5. I am committed to/enrolled in a plan to lose weight
6. I have lost weight in the past year and have been able to keep it off
7. I have lost weight in the past year, but have **not** been able to keep it off

**BMI 25+ (S13 > 25) FOR ALL COUNTRIES EXCEPT SINGAPORE, BMI 27+ (S13 > 27) FOR SINGAPORE**

**S23.** Have you spoken to your healthcare provider about a weight loss plan within the past 6 months?

1. Yes
2. No

**HAS SPOKEN TO HCP ABOUT WEIGHT LOSS PLAN IN PAST 6 MONTHS (S23/1)**

**S24.** Which of the following healthcare providers have you talked to about weight loss in the past 6 months?

*Please select all that apply.*

[RANDOMIZE]

1. My primary care physician
2. Dietitian
3. Obesity specialist
4. Psychologist or Psychiatrist
5. Other healthcare professional (a nurse, a physician who specializes in a certain condition, such as diabetes) [ANCHOR]

**FINAL QUOTA QUESTION**

**S100**

1. PERSON WITH OBESITY N = 1,000
   1. AGE 18+ (S3>17)
   2. LIVES IN ONE OF THE APAC COUNTRIES OF INTEREST (S5/NE99)
   3. NOT PREGNANT (S4/1 or (S4/2-3 AND S9/2))
   4. DOES NOT PARTICIPATE IN INTENSE FITNESS OR BODY BUILDING PROGRAMS (S10/2)
   5. CURRENT BMI 25+ (S13 ≥ 25) FOR ALL COUNTRIES EXCEPT SINGAPORE; CURRENT BMI 27+ (S13 ≥ 27) FOR SINGAPORE
   6. DID NOT HAVE SIGNIFICANT WEIGHT LOSS OR GAIN IN PAST (S17/2)
2. NOT QUALIFIED N=9999

**SOFT QUOTAS QUESTION**

**S102 COMMITTED TO ACTION**

1. COMMITTED TO ACTION N = 9999
   1. INTEND TO TAKE ACTION TO LOSE WT OR COMMITTED TO/ENROLLED IN PLAN TO LOSE WT (S22/4-5)
2. NOT COMMITTED TO ACTION N = 9999
   1. NOT ACTIVELY COMMITTED OR TAKING ACTION TO LOSE WEIGHT (S22/NE 4-5)

**SOFT QUOTAS QUESTION**

**S120 – MAINTAINING WEIGHT LOSS SUCCESS**

1. MAINTAINING Weight loss (S21/1) N=9999
2. NOT CURRENTLY MAINTAINING WEIGHT LOSS (S21/2) N=9999

**SOFT QUOTAS QUESTION**

**S125** WEIGHT CLASSIFICATION

[FOR ALL COUNTRIES EXCEPT SINGAPORE:

1. Obesity Class 1 (S13 ≥ 25 AND S13 < 30) N=9999
2. Obesity Class 2 (S13 ≥ 30 AND S13 < 35) N=9999
3. Obesity Class 3 (S13 ≥ 35 AND S13 < 40) N=9999
4. Obesity Class 4 (S13 ≥ 40) N=9999

[FOR SINGAPORE:

1. Obesity Class 1 (S13 ≥ 27 AND S13 < 32) N=9999
2. Obesity Class 2 (S13 ≥ 32 AND S13 < 37) N=9999
3. Obesity Class 3 (S13 ≥ 37 AND S13 < 41) N=9999
4. Obesity Class 4 (S13 ≥ 42) N=9999

**SECTION 100: Patient Weight History / Demographics**

**ALL RESPONDENTS (S100/1)**

**Q100.** You have qualified for the full survey. Thank you so much for your willingness to participate in this important research study – your input is very valuable! Please be aware that we may be asking some questions about your personal health.

We appreciate your open and honest feedback and want to assure you that your responses will be kept strictly confidential and only reported in summary with other respondents’ data.

Which of the following best describes your current employment status?

1. Employed full-time
2. Employed part-time
3. Self-employed
4. Not employed, but looking for work
5. Not employed and not looking for work
6. Retired
7. Student
8. Permanent disability
9. Other
10. Decline to answer

**ALL RESPONDENTS (S100/1)**

**Q101.** What is your marital status?

1. Married / Common-law or Live in Partner
2. Single
3. Divorced / Separated
4. Widowed
5. Other

**ALL RESPONDENTS (S100/1)**

**Q101A.** In general, would you say your health is:

[DO NOT SHOW #S ON SCREEN]

| Excellent  1 | Very Good  2 | Good  3 | Fair  4 | Poor  5 |  |
| --- | --- | --- | --- | --- | --- |

**ALL RESPONDENTS (S100/1)**

**Q103.** Which of the following do you believe best describes your current weight?

1. Underweight
2. Normal weight
3. Overweight
4. Obese
5. Extremely obese

**ALL RESPONDENTS (S100/1)**

**Q120.** Have you discussed your weight and/or talked about losing weight with a healthcare provider (physician, nurse, etc.) in the past 5 years?

1. Yes
2. No

**ALL RESPONDENTS (S100/1)**

**Q121A.** [IF DISCUSSED (Q120/1) “Which of the following healthcare providers have you ever discussed your weight with?

*Please consider all weight related conversations, regardless of who initiated the conversation*.”]

IF HASN’T DISCUSSED (Q120/2) “Which of the following healthcare providers would you consider discussing your weight with?”]

*Please select all that apply.*

1. My primary care physician
2. Dietitian or Nutritionist
3. Weight specialist
4. Psychologist or Psychiatrist
5. Other healthcare professional (a physician who specializes in a certain condition, such as diabetes) [ANCHOR]
6. [IF Q120/1 DISPLAY: None] [IF Q120/2 DISPLAY:I would not talk to a healthcare provider about my weight]

**ALL RESPONDENTS (S100/1)**

**Q122A.** Approximately how old were you when you first remember struggling with excess weight or obesity?

*Your best estimate will do.*

[RANGE 0-S3]

Age |_|_|

[IF DISCUSSED (Q120/1) ASK Q122. ELSE JUMP TO Q200]

**HAS DISCUSSED WITH HCP (Q120/1)**

**Q122.** Approximately how old were you when a healthcare provider first discussed your excess weight or recommended that you lose weight?

*Your best estimate will do.*

[RANGE Q122A-S3]

Age |_|_|

**SECTION 200: READINESS TO CHANGE / PREVIOUS SUCCESS**

**ALL RESPONDENTS (S100/1)**

**Q200.** Now, we would like to understand your overall goals concerning your weight and health. Please select the top 3 most important goals for you to personally achieve as part of your weight management, if any.

*Please select only 3 items.*

[SELECT 3] [RANDOMIZE]

1. Maintain current weight without gaining more
2. To lose (any amount of) weight
3. To lose a pre-specified % of my body weight
4. To lose a pre-specified number on the scale
5. To decrease the number of medications I must take
6. To improve my existing health condition(s)
7. To reduce the risks associated with excess weight / prevent a health condition
8. To have more energy
9. To improve my appearance
10. To buy / wear clothing that I like
11. To feel more confident / less judged by other people
12. To improve my sex life
13. To improve chances for career progression
14. To stay active with my family
15. To live a longer life
16. Other [ANCHOR]
17. None, I do not have any weight management goals [EXCLUSIVE] [ANCHOR]

HCP: Q225

**ALL RESPONDENTS (S100/1)**

**Q203.** Which of the following, if any, have motivated you the most to lose weight?

*Please select all that apply.*

[MULTIPLE RESPONSE] [RANDOMIZE WITHIN GROUP. RANDOMIZE GROUP ORDER, DON’T SHOW HEADINGS]

**Physical Health**

1. Having general health concerns
2. Wanting to stop or not need to take medication for a weight-related health condition
3. Reaching the upper end of the weight range I am comfortable with
4. A specific personal medical event (heart attack, stroke, etc.) or diagnosis (diabetes, liver disease, sleep apnea, etc.)

**Support**

1. Encouragement, support, recommendations from family or friends
2. Encouragement, support, recommendations from a healthcare provider
3. Encouragement, support, recommendations from wellness/fitness programs or a personal trainer
4. Encouragement, support from others who are trying to lose weight (losing weight with a spouse, work, health or fitness competition, etc.)

**Appearance**

1. Wanting to fit into a smaller clothing size
2. Wanting to be more fit/in better shape

**Goals**

1. Wanting to feel better physically, have more energy or be more active
2. Wanting to be more confident/improve my self-esteem
3. Wanting to improve my job performance
4. Wanting to improve my sex life
5. Wanting to be a positive role model for my family/children

**Life Events**

1. A major life change such as retirement, divorce, break-up, starting a family
2. An upcoming special occasion or event
3. A specific medical event (heart attack, stroke, etc.) or diagnosis (diabetes, liver disease, sleep apnea, etc.) in a family member/close friend
4. Other [ANCHOR]
5. None of the above/I have no desire to lose weight [EXCLUSIVE] [ANCHOR]

HCP: Q215

**ALL RESPONDENTS (S100/1)**

**Q205.** How many times in your adult life (after age 18) have you made a serious weight loss effort (e.g., followed a program, set goals, put your mind to it, or worked with a qualified healthcare professional), whether or not you were successful?

*Please enter 0 if you have never made a serious weight loss effort.*

*Please provide your best estimate.*

[RANGE 0-99]

# weight loss effort(s) |_|_|_|

[IF MADE weight loss effort (Q205/ >0) ASK Q210A. ELSE JUMP TO Q245.]

HCP: Q515

**MADE weight loss effort (Q205/ >0)**

**Q210A.** Which of the following methods for managing your weight have you ever **discussed** with a healthcare provider?

*Please select all that apply.*

[MULTI-SELECT, RANDOMIZE ROWS WITHIN GROUP, RANDOMIZE GROUP ORDER EXCEPT 16 and 99 ITEMS, DON’T SHOW HEADINGS]

**Diet / Healthy Eating**

1. General improvement in eating habits / reducing calories
2. Specific diet or diet program [FOR INDONESIA DISPLAY: (e.g. Keto diet, intermittent fasting, OCD diet, Mayo diet, high protein diet, Mediterranean diet, mesotherapy)] [FOR INDIA DISPLAY: (e.g. Keto diet, intermittent fasting, vegan diet, low carb)] [FOR PAKISTAN DISPLAY: (e.g. Keto diet, Atkins, Mediterranean diet)] [FOR MALAYSIA DISPLAY: (e.g. plate method, meal replacement program, low glycemic index, moderate carb diet)] [FOR SINGAPORE DISPLAY: (e.g. Low-carb diet, Keto diet, intermittent fasting, fixed caloric deficit, very low calorie or low calorie diet with meal replacements, time-restricted feeding, low-fat diet)] [FOR PHILIPPINES DISPLAY: (e.g. Calorie Counting, Intermittent Fasting, Vegan/Vegetarian, Paleo, Ketogenic diet, low-fat, South Beach diet, gluten free diet, Blood type diet] [FOR THAILAND, VIETNAM AND BANGLADESH DISPLAY: (e.g. Keto, low-carb, low-fat, intermittent fasting)]
3. Elimination diets

**Exercise**

1. Generally, be more active / increase physical activity
2. A formal exercise program / gym membership / personal trainer

**Tracking**

1. Meal / nutrient tracking (on paper or an app)
2. Exercise tracking (on paper or app such as smartphone apps, wearable fitness tracker, etc.)

**Medical Treatment / Medication**

1. Over-the-counter (non-prescription) weight loss medication (vitamins, supplements, etc.)
2. Prescription weight loss medication
3. Visiting a nutritionist / dietitian (non-physician)
4. Visiting an obesity specialist
5. Behavior therapy or psychotherapy such as counseling or behavior modification
6. Weight loss surgery / bariatric surgery

**Quality of life management**

1. Stress management
2. Sleep quality management
3. Other [ANCHOR]
4. None of the above [EXCLUSIVE][ANCHOR]

**DISCUSSED ANY METHOD OF MANAGING WEIGHT WITH AN HCP (Q210A/ne 99)**

**Q211.** For each of these methods for managing your weight you have **ever discussed** with a healthcare provider, which type of healthcare provider did you have the discussion with?

[display as grid with hcp types in columns, codes selected at q210a as rows]

[PN: ALLOW FOR MULTIPLE SELECTIONS PER ROW; DO NOT RECALL IS EXCLUSIVE IN ROW]

[DISPLAY ROWS IN SAME ORDER AS q210a, DON’T SHOW HEADINGS]

[COLUMN HEADERS: Primary Care Physician / General Practitioner Obesity specialist Other Specialist Do Not Recall

**Diet / Healthy Eating**

1. General improvement in eating habits / reducing calories
2. Specific diet or diet program [FOR INDONESIA DISPLAY: (e.g. Keto diet, intermittent fasting, OCD diet, Mayo diet, high protein diet, Mediterranean diet, mesotherapy)] [FOR INDIA DISPLAY: (e.g. Keto diet, intermittent fasting, vegan diet, low carb)] [FOR PAKISTAN DISPLAY: (e.g. Keto diet, Atkins, Mediterranean diet)] [FOR MALAYSIA DISPLAY: (e.g. plate method, meal replacement program, low glycemic index, moderate carb diet)] [FOR SINGAPORE DISPLAY: (e.g. Low-carb diet, Keto diet, intermittent fasting, fixed caloric deficit, very low calorie or low calorie diet with meal replacements, time-restricted feeding, low-fat diet)] [FOR PHILIPPINES DISPLAY: (e.g. Calorie Counting, Intermittent Fasting, Vegan/Vegetarian, Paleo, Ketogenic diet, low-fat, South Beach diet, gluten free diet, Blood type diet] [FOR THAILAND, VIETNAM AND BANGLADESH DISPLAY: (e.g. Keto, low-carb, low-fat, intermittent fasting)]
3. Elimination diets

**Exercise**

1. Generally, be more active / increase physical activity
2. A formal exercise program / gym membership / personal trainer

**Tracking**

1. Meal / nutrient tracking (on paper or an app)
2. Exercise tracking (on paper or app such as smartphone apps, wearable fitness tracker, etc.)

**Medical Treatment / Medication**

1. Over-the-counter (non-prescription) weight loss medication (vitamins, supplements, etc.)
2. Prescription weight loss medication
3. Visiting a nutritionist / dietitian (non-physician)
4. Visiting an obesity specialist
5. Behavior therapy or psychotherapy such as counseling or behavior modification
6. Weight loss surgery / bariatric surgery

**Quality of life management**

1. Stress management
2. Sleep quality management
3. Other [ANCHOR]
4. None of the above [EXCLUSIVE][ANCHOR]

**MADE weight loss effort (Q205/ >0)**

**Q210C.** Which of the following methods for managing your weight **are you currently trying**?

*Please select all that apply.*

[INSERT FULL LIST FROM Q210A. SHOW IN SAME ORDER.]

99. [ ] I am not currently trying any of these methods [EXCLUSIVE]

**MADE weight loss effort (Q205/ >0)**

**Q210D.** Which of the following methods for managing your weight do you think are **effective for weight loss**?

*Please select all that apply.*

[INSERT LIST FROM Q210A. SHOW IN SAME ORDER.]

Diet / Healthy Eating

1. General improvement in eating habits / reducing calories
2. Specific diet or diet program [FOR INDONESIA DISPLAY: (e.g. Keto diet, intermittent fasting, OCD diet, Mayo diet, high protein diet, Mediterranean diet, mesotherapy)] [FOR INDIA DISPLAY: (e.g. Keto diet, intermittent fasting, vegan diet, low carb)] [FOR PAKISTAN DISPLAY: (e.g. Keto diet, Atkins, Mediterranean diet)] [FOR MALAYSIA DISPLAY: (e.g. plate method, meal replacement program, low glycemic index, moderate carb diet)] [FOR SINGAPORE DISPLAY: (e.g. Low-carb diet, Keto diet, intermittent fasting, fixed caloric deficit, very low calorie or low calorie diet with meal replacements, time-restricted feeding, low-fat diet)] [FOR PHILIPPINES DISPLAY: (e.g. Calorie Counting, Intermittent Fasting, Vegan/Vegetarian, Paleo, Ketogenic diet, low-fat, South Beach diet, gluten free diet, Blood type diet] [FOR THAILAND, VIETNAM AND BANGLADESH DISPLAY: (e.g. Keto, low-carb, low-fat, intermittent fasting)]
3. Elimination diets

Exercise

1. Generally, be more active / increase physical activity
2. A formal exercise program / gym membership / personal trainer

Tracking

1. Meal / nutrient tracking (on paper or an app)
2. Exercise tracking (on paper or app such as smartphone apps, wearable fitness tracker, etc.)

Medical Treatment / Medication

1. Over-the-counter (non-prescription) weight loss medication (vitamins, supplements, etc.)
2. Prescription weight loss medication
3. Visiting a nutritionist / dietitian (non-physician)
4. Visiting an obesity specialist
5. Behavior therapy or psychotherapy such as counseling or behavior modification
6. Weight loss surgery / bariatric surgery

Quality of life management

1. Stress management
2. Sleep quality management
3. Other [ANCHOR]

99 None of the above [EXCLUSIVE][ANCHOR]

**MADE weight loss effort (Q205/ >0)**

**Q213.** Have you ever had what you’d consider a successful weight loss effort, but later regained the weight after keeping it off for at least 6 months?

1. Yes
2. No

[IF REGAINED WEIGHT (Q213/1) ASK Q214]

**REGAINED WEIGHT (Q213/1)**

**Q214.** Which of the following do you feel most contributed to regaining weight after your weight loss effort?

*Please select all that apply.*

[RANDOMIZE]

1. It was difficult to stay motivated
2. I no longer followed my eating plan after losing the weight
3. I no longer exercised after losing the weight
4. I stopped taking a weight loss medication [ONLY SHOW IF SELECT Q210C/8-9 OR Q210D/8-9]
5. A major life change (e.g. change of residence, death of relative, etc.)
6. It was difficult to maintain the changes I had made to lose the weight
7. I no longer felt the weight loss was worth the effort
8. Medical reasons (e.g., thyroid problems, new medication caused weight gain)
9. Cost of treatment options
10. Cost of wellness/fitness programs/memberships or personal trainers
11. My job demands [SHOW IF EMPLOYED (Q100/1-3)]
12. Other [ANCHOR]

**ALL RESPONDENTS (S100/1)**

**Q245B.** As an outcome of a weight loss effort, what weight would you set for yourself as a goal?

[RANGE 1-999]

Kilograms: |_|_|_|

**ALL RESPONDENTS (S100/1)**

**Q245C** HIDDEN CALCULATION FOR % WEIGHT LOSS

[Q245C = (100 * S12 – Q245B) / S12]

**ALL RESPONDENTS (S100/1)**

**Q246.** How much do you agree with the following statements?

| Strongly disagree  1 | Disagree  2 | Neutral  3 | Agree  4 | Strongly agree  5 |
| --- | --- | --- | --- | --- |

[RANDOMIZE, DON’T SHOW HEADINGS]

**COPD Helplessness Index - ADAPTED**

1. No matter what I do or how hard I try, I just can’t seem to overcome my weight issues
2. When it comes to managing my weight, I feel I can only do what my physician tells me to do
3. My weight is controlling my life
4. It seems as though fate and other factors beyond my control affect my weight

**Dietary Helplessness and Disinhibition in Weight**

1. No matter how hard I try to change, I end up falling back into some of my old eating habits

**SECTION 400: OBESITY AWARENESS AND PERCEPTIONS**

HCP: Q405

**ALL RESPONDENTS (S100/1)**

**Q405.** In general, how large of an impact do you believe the following health conditions have on a person’s overall health?

*Use a scale where 1 means “Very little impact” and 5 means “An extreme impact.”*

*1 - Very little impact 2 3 4 5 - An extreme impact 99 - Not familiar with this condition*

[RANDOMIZE]

1. Diabetes
2. Chronic obstructive pulmonary disease (COPD)
3. Cancer
4. Stroke
5. Obesity [FOR ALL COUNTRIES EXCEPT SINGAPORE DISPLAY: (BMI of 25 or greater)] [FOR SINGAPORE DISPLAY: (BMI of 27 or greater)]

**ALL RESPONDENTS**

**Q405A** HIDDEN QUESTION

[RECODE Q405 ATTRIBUTES 1-4 BASED ON IF THEY’RE GREATER OR LESS THAN OBESITY (Q405_5)]

OBESITY IS MORE SERIOUS (Q405_ATTRIBUTE < Q405_5)

OBESITY IS AS SERIOUS (Q405_ATTRIBUTE = Q405_5)

OBESITY IS LESS SERIOUS (Q405_ATTRIBUTE > Q405_5)

**ALL RESPONDENTS (S100/1)**

**Q415.** Assuming you remain at your current weight, how much do you worry that your weight may affect your health in the future?

1. Not at all
2. A little
3. Somewhat
4. A lot
5. An extreme amount

**ALL RESPONDENTS (S100/1)**

**Q420** Compared to a person who does not have obesity, how easy or difficult do you think each of the following is for someone who has obesity?

| [RANDOMIZE] | Much harder | Somewhat harder | About the same | Somewhat easier | Much easier |
| --- | --- | --- | --- | --- | --- |
| Getting a job | 1 | 2 | 3 | 4 | 5 |
| Advancing/promotion in a job | 1 | 2 | 3 | 4 | 5 |
| Making friends | 1 | 2 | 3 | 4 | 5 |
| Forming romantic relationships | 1 | 2 | 3 | 4 | 5 |

**ALL RESPONDENTS (S100/1)**

**Q425** How much of an impact do you think a person having obesity has on each of the following?

| [RANDOMIZE] | Very negative impact | Somewhat negative impact | No impact | Somewhat positive impact | Very positive impact |
| --- | --- | --- | --- | --- | --- |
| How smart people think the person is | 1 | 2 | 3 | 4 | 5 |
| How athletic people think the person is | 1 | 2 | 3 | 4 | 5 |
| How healthy people think the person is | 1 | 2 | 3 | 4 | 5 |
| Relationships with friends | 1 | 2 | 3 | 4 | 5 |
| Relationships at home/with family | 1 | 2 | 3 | 4 | 5 |
| How much ambition people think the person has | 1 | 2 | 3 | 4 | 5 |
| How much willpower people think the person has | 1 | 2 | 3 | 4 | 5 |

**ALL RESPONDENTS (S100/1)**

**Q435.** For quality control purposes, please select No.

1. Yes
2. No
3. Maybe

**SECTION 500: OBESITY ATTITUDINAL QUESTIONS**

HCP: Q503

**ALL RESPONDENTS (S100/1)**

**Q500.** Please indicate how much you agree with each of the following…

*Use a scale where 1 means “Do not agree at all” and 5 means “Completely agree.”*

*1 - Do not agree at all 2 3 4 5 - Completely agree*

[RANDOMIZE, CAROUSEL]

1. It is easy for me to lose weight.
2. I could lose weight if I really set my mind to it.
3. If I lost weight, it would be easy for me to keep the weight off.
4. I know how to lose weight.
5. My healthcare provider has a responsibility to actively contribute to a successful weight loss effort.
6. My weight loss is completely my responsibility.
7. For me to lose weight, I would need to completely change my lifestyle.
8. I am happy with my current weight.
9. I am past the point where I can lose weight on my own.
10. I am motivated to lose weight.
11. For quality control purposes, please select 1.
12. Obesity is less important to me than other diseases.
13. I do not feel comfortable bringing up my weight unless my healthcare provider mentions it first.
14. There is nothing my doctor can do to help me manage my weight.
15. I know how to keep the weight off. [SHOW AFTER CODE 4]

HCP: Q507

**ALL RESPONDENTS (S100/1)**

**Q507.** How much do you agree that each of the following is a barrier to you losing weight?

*Please use a scale where 1 means “Do not agree at all” and 5 means “Completely agree.”*

1 - Do not agree at all 2 3 4 5 - Completely agree

[RANDOMIZE, CAROUSEL]

1. My preference for unhealthy food
2. Lack of exercise
3. My genes (e.g., inherited from my family)
4. The nature of my job / employment [SHOW IF EMPLOYED (Q100/1-3)]
5. A lack of time to cook healthy meals
6. My other health conditions
7. My friends and family
8. My healthcare provider
9. My finances
10. My lack of motivation
11. My lack of ability to control my hunger
12. The cost of healthy food
13. Limited access to healthy food
14. My mental health / emotional status
15. Fear of failure
16. Limited coverage for health care costs
17. Limited mobility due to physical health problems
18. The possibility of regaining the weight
19. My unhealthy eating habits (large portion sizes, excessive snacking)
20. My lack of understanding of what obesity is
21. The cost of weight management medications, programs and services
22. My metabolism
23. My age
24. High carbohydrate diet

HCP: Q521

**ALL RESPONDENTS (S100/1)**

**Q520.** Please indicate how much you agree with the following regarding prescription medications for weight loss…

*Use a scale where 1 means “Do not agree at all” and 5 means “Completely agree.”*

[CAROUSEL]

[KEEP ROW ITEMS 8 AND 9 TOGETHER]

1 - Do not agree at all 2 3 4 5 - Completely agree 9 Don’t know/ Not sure

1. A weight loss medication available by prescription from my physician would be more effective than other treatment options for weight loss.
2. If I heard of a new prescription weight loss medication, I would ask my physician to prescribe it to me.
3. I would rather take a prescription medication than have a surgery (bariatric) to lose weight.
4. I am concerned about the side effects associated with prescription weight loss medications.
5. I would like my healthcare provider to offer me a prescription weight loss medication to help me with my weight loss efforts.
6. I trust my healthcare provider to recommend a prescription weight loss medication that is right for me.
7. There are good options available today for prescription weight loss medications.
8. Cost is a major barrier for me to consider using prescription weight loss medications.
9. I would rather lose weight myself than depend on medication.
10. I am concerned about the long-term safety associated with prescription weight loss medications.

HCP: Q525

**ALL RESPONDENTS (S100/1)**

**Q525.** Please indicate how much you agree with the following regarding weight loss surgery…

*Note that weight loss surgery is also known as bariatric surgery.*

*Use a scale where 1 means “Do not agree at all” and 5 means “Completely agree”*

[CAROUSEL]

1 - Do not agree at all 2 3 4 5 - Completely agree 99 Don’t know/ Not sure

1. Weight loss surgery is more effective than other treatment options for weight loss.
2. I would rather have weight loss surgery than change my lifestyle to lose weight.
3. I have concerns about the safety of having weight loss surgery.
4. I trust my healthcare provider to recommend weight loss surgery if it is right for me.
5. There are good options available today for weight loss surgery.
6. Cost is a major barrier for me when considering weight loss surgery.
7. My healthcare provider is likely to review weight loss surgery options with me.
8. The wait time for weight loss surgery is too long.
9. Having weight loss surgery also means having a permanent change in lifestyle.
10. I would rather lose weight with diet and exercise than have weight loss surgery.
11. Having weight loss surgery is the “easy” way out.
12. After weight loss surgery, the weight may come back.
13. Weight loss surgery is a last option after failed attempts with lifestyle and anti-obesity medications.

**SECTION 600: SUPPORT STRUCTURE**

**ALL RESPONDENTS (S100/1)**

**Q600.** Which of the following have you used as a source of information for managing your weight?

*Please select all that apply.*

[RANDOM, MULTI-SELECT]

1. The Internet [DISPLAY FOR INDIA, INDONESIA, THAILAND, BANGLADESH:(Google, social media, websites)] [DISPLAY FOR PAKISTAN, SINGAPORE, VIETNAM: (Google, Yahoo, social media, websites)] [DISPLAY FOR MALAYSIA: (Google, Yahoo, Bing, Petal Search, social media, websites)] [DISPLAY FOR PHILIPPINES: (Google, Safari, Bing, Yahoo, social media, websites)]
2. Family and friends
3. Information from a healthcare provider
4. [REMOVED]
5. Wellness coach or personal trainer
6. Books or magazines
7. [REMOVED]
8. Weight loss programs
9. Dietitian or nutritionist (non-physician)
10. [REMOVED]
11. Peer support group
12. [REMOVED]
13. [REMOVED]
14. [REMOVED]
15. Smartphone apps
16. [REMOVED]
17. Television programs
18. None of the above [ANCHOR, EXCLUSIVE]

HCP: Q650

**ALL RESPONDENTS (S100/1)**

**Q640.** Please indicate how much you agree with the following statements regarding obesity and weight management.

*Use a scale where 1 means “Do not agree at all” and 5 means “Completely agree.”*

1 - Do not agree at all 2 3 4 5 - Completely agree 99 - Does not apply

[RANDOMIZE]

1. Maintaining a healthy weight is a priority for our country’s healthcare system.
2. Cost of obesity therapy / treatment is a barrier to me losing weight.
3. I feel the healthcare system (doctor’s offices, hospitals, etc.) is a good resource for those looking to lose weight.
4. [DISPLAY IF CURRENTLY EMPLOYED but not self-employed (Q100/1-2)] My employer is an important partner in my efforts to manage my weight.
5. Obesity is a chronic disease.
6. A loss of 5-10% body weight would be extremely beneficial to my overall health.
7. The treatment of obesity should be a team effort between different medical professionals.
8. For quality control purposes, please select 3.

**SECTION 700: INTERACTION WITH HCP**

[IF HAS DISCUSSED EXCESS/LOSING WEIGHT WITH AN HCP (Q120/1) ASK Q700. ELSE JUMP TO Q702.]

**HAS DISCUSSED EXCESS/LOSING WEIGHT WITH HCP (Q120/1)**

**Q700.** Have you ever been diagnosed with obesity by a medical doctor or qualified healthcare professional?

1. Yes
2. No

HCP: Q702

**HAS DISCUSSED EXCESS/LOSING WEIGHT WITH HCP (Q120/1)**

**Q701.** Who typically brings up your weight during your appointments?

1. I usually start the conversation.
2. My healthcare provider usually starts the conversation.

HCP: Q702

**ALL RESPONDENTS (S100/1)**

**Q702.** [IF HCP HAS BROUGHT UP WEIGHT (Q701/2) “Do you like that your healthcare provider brings up your weight during appointments?”]

[IF HCP HAS NOT BROUGHT UP WEIGHT (Q701/NE2 OR Q120/2) “Would you like for your healthcare provider to bring up your weight during appointments?”]

1. Yes
2. No

HCP: Q725

**HAS DISCUSSED WEIGHT WITH HCP (Q120/1)**

**Q710.** Thinking about your most recent discussion, how did you feel after discussing your weight with your healthcare provider?

*Please select all that apply.*

[MULTI-SELECT] [RANDOMIZE]

1. Motivated
2. Hopeful
3. Supported
4. Embarrassed
5. Discouraged
6. Blamed
7. Offended
8. Confused
9. Relieved
10. Indifferent
11. Rushed
12. Other [ANCHOR]

HCP: Q720

**ALL RESPONDENTS (S100/1)**

**Q720.** [IF DISCUSSED (Q120/1) “What types of weight management goals have you set with your healthcare provider?”]

IF HASN’T DISCUSSED (Q120/2) “What types of weight management goals would you like to set with your healthcare provider?”]

*Please select all that apply.*

[RANDOMIZE]

1. To not gain any more weight
2. To lose weight (did not specify an amount)
3. To lose a pre-specified % of my body weight
4. To lose a pre-specified number on the scale
5. To decrease the number of medications I must take
6. To improve my existing health condition(s)
7. To reduce the risks associated with weight / prevent a health condition
8. To have more energy
9. To improve my appearance
10. Short-term (within the next 6 months) weight loss goals
11. Long-term (more than six months from now) weight loss goals
12. To improve my lifestyle
13. To reduce my stress and improve overall health and well-being
14. To improve my physical and mental health and well-being
15. Other [ANCHOR]
16. [IF DISCUSSED (Q120/1) “I have not set any goals with my healthcare provider” IF HASN’T DISCUSSED (Q120/2) “I would not like to set any goals with my healthcare provider”] [EXCLUSIVE][ANCHOR]

IF DISCUSSED AND HCP RECOMMENDED LOSS OF percent body weight (Q120/1 AND Q720/3) ASK Q725. ELSE JUMP TO PN BEFORE Q726]

**DISCUSSED AND HCP RECOMMENDED LOSS OF percent body weight (Q120/1 AND Q720/3)**

**Q725.** You mentioned your healthcare provider suggested you lose a percentage of your body weight., What percent did they suggest you try to lose?

[Range 1-100]

Percent of body weight |_|_|_|%

IF DISCUSSED AND HCP RECOMMENDED LOSING WEIGHT (Q120/1 AND Q720/4) ASK Q726. ELSE JUMP TO Q742]

**DISCUSSED AND HCP RECOMMENDED TARGET WEIGHT LOSS (Q120/1) AND Q720/4)**

**Q726.** You mentioned your healthcare provider suggested you lose a certain number of kilograms. How many kilograms did they suggest you try to lose?

[RANGE 1-999]

Kilograms: |_|_|_|

**ALL RESPONDENTS (S100/1)**

**Q742.** Please indicate how much you agree with the following statements:

*Use a scale where 1 means “Do not agree at all” and 5 means “Completely agree.”*

1 - Do not agree at all 2 3 4 5 - Completely agree

[RANDOMIZE] [SHOW 1-6 IF DISCUSSED (Q120/1]

1. I feel comfortable talking to my healthcare provider about my weight.
2. My healthcare provider listens carefully to what I have to say about my weight.
3. My healthcare provider understands the difficulties of weight management.
4. I trust my healthcare provider’s advice when it comes to weight management.
5. I follow my healthcare provider’s advice about weight management.
6. It is important to me that my healthcare provider is at a healthy weight.

[RANDOMIZE] [SHOW 7-12 IF HASN’T DISCUSSED (Q120/2]

1. I would feel comfortable talking to my healthcare provider about my weight.
2. I expect my healthcare provider would listen carefully to what I have to say about my weight
3. I expect my healthcare provider would understand the difficulties of weight management.
4. I would trust my healthcare provider’s advice when it comes to weight management.
5. I would follow my healthcare provider’s advice about weight management.
6. It is important to me that my healthcare provider is at a healthy weight.

HCP: Q740

**ALL RESPONDENTS (S100/1)**

**Q759.** [IF DISCUSSED (Q120/1) “Did your healthcare provider schedule a follow-up appointment or call related to your weight after your last visit?”]

[IF HASN’T DISCUSSED (Q120/2) “Would you like your healthcare provider to schedule a follow-up appointment or call related to your weight after you visit?”]

1. Yes
2. No

HCP: Q708

**ALL RESPONDENTS (S100/1)**

**Q770.** Which of the following are/would be the **top five** reasons for which you **might not** discuss managing your weight with your healthcare provider?

*Please select up to 5 items only.*

[MULTISELECT 5 ANSWERS, RANDOMIZE]

1. The appointment is not long enough / I’m rushed
2. There are more important health issues / concerns to discuss
3. I do not feel comfortable bringing it up
4. I do not trust and/or do not have a close relationship with my healthcare provider
5. I do not see my weight as a significant medical issue
6. I am in good health and do not have weight-related health problems
7. I believe it is my responsibility to manage my weight
8. I am not interested in losing weight
9. I do not feel motivated to lose weight
10. I do not believe I am able to lose weight
11. Even if I were to lose weight, I would just gain it back
12. I already know what I need to do to manage my weight
13. There is nothing my healthcare provider can do to help me manage my weight
14. I do not think my healthcare provider is interested in / concerned about my weight
15. I have had previous bad experience discussing weight with a healthcare provider
16. I do not have the financial means to support a weight loss effort
17. My healthcare provider does not have training to provide weight management services
18. My healthcare provider’s office is not set up to treat patients with excess weight / obesity
19. Other [ANCHOR]

**SECTION 800: SOLUTION REVIEW**

HCP: Q760

**ALL RESPONDENTS (S100/1)**

**Q825.** Please select top 3 types of information that would be most helpful for you personally in managing your weight.

*Please select only three items.*

**Information on…**

[SELECT 3 ONLY]

[RANDOMIZE]

1. Healthy ways to lose weight
2. The health benefits of weight loss
3. Medical treatment options for weight management
4. Managing weight with exercise
5. How to maintain weight loss
6. How healthcare providers can help with weight management
7. Stress management techniques
8. Realistic weight loss goals
9. Exercises which are safe to do for people with mobility limitations
10. Healthy vs. non-healthy eating

HCP: Q602

**ALL RESPONDENTS (S100/1)**

**Q826.** What are the **top 5 types of support that would be most helpful for you personally** to be successful with managing your weight?

*Select your top 5.*

[5 SELECTIONS] [RANDOMIZE]

1. Resources for family and friends to help understand how to be supportive
2. Specific meal plans to follow for weight management
3. Online support groups for those trying to lose weight
4. Local in-person support groups for those trying to lose weight
5. Motivational programs to help people stay on track with weight loss plan
6. More programs offered at work to help people lose weight
7. Encouragement from friends/family to increase desire to keep going
8. Financial support for healthy choices (gym membership, healthy foods)
9. Diary for tracking weight over time (paper based or electronic)
10. Diary for tracking food intake (paper based or electronic)
11. Diary for tracking physical activity (paper based or electronic)
12. App with weight loss tracking and ideas for healthy eating and physical activity
13. Programs for physical activity
14. Prescription drugs for weight loss
15. Over-the-counter drugs for weight loss
16. Personal trainer / weight loss counselor
17. Weekly follow-up with a healthcare provider
18. Meetings with dietitian / nutritionist (non-physician)
19. A work culture that encourages a healthy lifestyle
20. Access to mental health support
21. Access to stress management support
22. Access to a physician who specializes in obesity
23. Other [ANCHOR]
24. I don’t need any of these types of support [EXCLUSIVE, ANCHOR]

**SECTION 900: DEMOGRAPHICS**

HCP: Q906

**ALL RESPONDENTS (S100/1)**

**Q900.** Thank you again for your time so far. As we noted at the beginning of this survey, your personal information will never be shared with other organizations for any purpose. Your honest answers are very much appreciated. Your responses to this survey will help the sponsor (Novo Nordisk) understand the healthcare experiences of patients.

To finish, we would like to gather some additional information used for categorization purposes.

Which of the following medical conditions have you ever been diagnosed with by a healthcare provider?

*Please select all that apply.*

[ALPHA SORT]

1. Cardiovascular Diseases (e.g. coronary heart disease, congestive heart failure, atrial fibrillation, pulmonary embolism, stroke)
2. Depression/Anxiety
3. High cholesterol (Dyslipidemia / triglycerides)
4. High blood pressure (Hypertension)
5. Infertility
6. Liver disease (e.g., Non-alcoholic fatty liver disease)
7. Obstructive Sleep Apnea
8. Osteoarthritis
9. Metabolic syndrome
10. Stomach or intestinal problems
11. Pre-diabetes
12. Diabetes (Type II)
13. Cancer
14. Polycystic Ovary Syndrome (PCOS) [ONLY SHOW IF FEMALE OR OTHER (S5/2-3)]
15. Eating disorder (e.g. binge eating disorder, night eating syndrome)
16. Other condition [ANCHOR]
17. None of these [EXCLUSIVE] [ANCHOR]

**ALL RESPONDENTS (S100/1)**

**Q930.** Have you ever had bariatric / weight loss surgery?

1. Yes
2. No

**EVER HAD BARIATRIC SURGERY (Q930/1)**

**Q932.** How many years ago did you have bariatric / weight loss surgery?

*If you had bariatric surgery less than one year ago, please enter “1” (one)*

[RANGE 1-S3]

|_|_|_| # of years ago had bariatric / weight loss surgery

**ALL RESPONDENTS (S100/1)**

**Q901.** How frequently do you weigh yourself?

*Please select the answer that best applies.*

1. Every day
2. 2-3 times a week
3. Once a week
4. Once every two weeks
5. Once a month
6. Once every two months
7. A few times a year
8. Never

**ALL RESPONDENTS (S100/1)**

**Q902.** In a typical week, how frequently do you exercise for at least a 20-minute period?

1. Never
2. Less than once a week
3. 1 to 2 times a week
4. 3 to 4 times a week
5. 5 to 6 times a week
6. 7 or more times a week

HCP: Q920

**ALL RESPONDENTS (S100/1)**

**Q929.** Which of the following best describes the size of the area in which you live?

1. Urban area
2. Suburban area close to a city
3. Rural area / village
