# Supplementary material for: Perceptions, attitudes, behaviours and barriers towards obesity among people with obesity and health care professionals in Indonesia: An exploratory online survey
Source: PLoS One. 2026 Jun 4;21(6):e0350857. doi: 10.1371/journal.pone.0350857 (PMC13235876; doi:10.1371/journal.pone.0350857)
Supplement: S2 File — (DOCX) [file pone.0350857.s005.docx]

KJT Group, Inc.

6 East St

Honeoye Falls, NY, 14472

**Awareness, Care & Treatment In Obesity MaNagement ACTION APAC - HCP Questionnaire**

**KJT Contacts**

| Lynn Clement  Email: lynn@kjtgroup.com  Phone: 585-624-8050 x304 | Andrea Stoltz  Email: andreas@kjtgroup.com  Phone: 585-624-8050 x333 |  |  |
| --- | --- | --- | --- |
| Nick Henderson  Email: nickh@kjtgroup.com  Phone: 585-624-8050 x348  Rebecca Hahn  Email: rebeccah@kjtgroup.com  Phone: 585-624-8050 x312 | Peg Jaynes  Email: pegj@kjtgroup.com  Phone: 585-624-8050 x353 |  |  |

**Recruitment:**

|  | **Indonesia** | **India** | **Pakistan** | **Thailand** | **Malaysia** | **Singapore** | **Philippines** | **Vietnam** | **Bangladesh** | **TOTAL** |
| --- | --- | --- | --- | --- | --- | --- | --- | --- | --- | --- |
| **HCPs** | **200** | **300** | **200** | **200** | **200** | **200** | **200** | **200** | **200** | **1900** |
| **PCPs** | 100 | 150 | 100 | 100 | 100 | 100 | 100 | 100 | 100 | 950 |
| **Specialists** | 100 | 150 | 100 | 100 | 100 | 100 | 100 | 100 | 100 | 950 |

**Screening Criteria:**

**HCPs:**

- Physician
- Specialty is **NOT** plastic surgeon, general surgeon, or bariatric surgeon
- Spends at least 50% of time in patient medical management (as opposed to surgical procedures, office procedures or research/administrative tasks)
- Practices in one of the 9 countries
- In practice 2+ years
- Has seen at least 100 adult patients in past month
- Has seen at least 10 adult patients in past month who have obesity: defined as a patient with a Body Mass Index (BMI) ≥25 with or without comorbidities (do NOT need to be treating patients for obesity, only seen them) (for Singapore only BMI of ≥27

**Specialty Quota (Hard Quota):**

- **Primary Care Providers**
  - Specialty is Family Practice, General Practice, Internal Medicine
- **Non-PCP Specialties**
  - Specialty is , Endocrinologist, Cardiologist, Gastroenterologist, Obstetrics/Gynecologist, Bariatrics/Obesity medicine, Nutrition Specialist (physician), Orthopaedist, Aesthetics Medicine.

**Obesity Specialist Definition (will fall out naturally):**

- 50% or more of patients are seen for obesity/weight management (S35)

Data Validation Questions:

**Q240, Q503_11, Q650_8**

**SECTION S: SCREENER**

**ALL RESPONDENTS**

**S0** Thank you for taking the time to participate in this important research. For your convenience we are offering this survey in multiple languages. Please select a language that you are most familiar with.

1. English [DISPLAY FOR ALL]
2. Bengali / Bangla [DISPLAY FOR BANGLADESH]
3. Hindi [DISPLAY FOR INDIA AND PAKISTAN]
4. Indonesian [DISPLAY FOR INDONESIA]
5. Bahasa Malaysia / Malay [DISPLAY FOR MALAYSIA]
6. Urdu [DISPLAY FOR PAKISTAN]
7. Tagalog / Filipino [DISPLAY PHILIPPINES]
8. Malay [DISPLAY FOR SINGAPORE]
9. Thai [DISPLAY FOR THAILAND]
10. Vietnamese [DISPLAY FOR VIETNAM]
11. Standard Chinese (Mandarin) [DISPLAY FOR SINGAPORE, MALAYSIA]
12. Tamil [DISPLAY FOR SINGAPORE, MALAYSIA]

**ALL RESPONDENTS**

**S1**. Thank you for your interest in this survey. We appreciate your willingness to participate in this important research on healthcare issues.

Before participating, KJT Group requires you to review the following information:

- KJT Group is a **global market research company** requesting your consent to participate in this research survey. KJT Group is the data controller.
- KJT’s legal basis for the processing of your personal data is your consent.
- Your participation is fully voluntary; you can choose to stop at any time and you can, after completion of the questionnaire, withdraw your consent at any time by writing to [help@kjtgroup.com](mailto:help@kjtgroup.com) without any penalty or loss of benefits to which you are otherwise entitled.
- The purpose of the survey is to help the sponsor (Novo Nordisk) understand the healthcare experiences of patients.The risk of the research is to your privacy. Your responses will be kept **strictly confidential** and will never be associated with your name. Your alternative is to not participate in the survey.
- You have the **right to see and get a copy your data, amend your data, or erase your data** at any time.
- We expect, on average, it will take respondents like yourself **20 minutes** to complete this survey.
- Your responses will be aggregated with other responses in KJT Group’s research report and will be made publicly available in a peer-reviewed scientific journal publication once the study has finished. However, **your name will never be included in the report, publication, or identified to the sponsor**.
- Your responses will be transferred to and **stored on secure servers in the United States**.
- We will **destroy your personally identifiable information within 12 months of data collection**; however, we will maintain a permanent record of this consent.
- If you do not qualify for the study, your personal data will be stored electronically by KJT Group and erased no later than 12 months after the end of data collection.
- All information from this study will be stored for at least 5 years after the Study Report is made, or according to local requirements. The study report contains the full results of the study.
- Your personal information is protected by the data protection law as applicable in your country.
- Your **participation is voluntary,** and you may choose to stop participating at any time (withdraw consent) without any penalty or loss of benefits to which you are otherwise entitled.
- If you qualify and complete this survey, you will receive online panel credit/the honoraria listed in your invitation. There are no costs to you for your participation.
- KJT Group will make sure the study information we have collected about you cannot be looked at by people who are not authorized to do so. To make sure the study is done correctly and to check the results, the following people will be able to see your study information:
  - The Research Ethics Committee/Institutional Review Board
  - National medicine authorities from other countries
- A description of this clinical trial will be available on http://www.ClinicalTrials.gov, as required by U.S. Law. This Web site will not include information that can identify you. At most, the Web site will include a summary of the results. You can search this Web site at any time.

You may contact 585-624-8050 (24 hours) or [help@kjtgroup.com](mailto:help@kjtgroup.com) with questions or concerns, or if you would like to follow-up on these points or need additional support.

• Protocol number: DAS-006

• IRB Protocol number: 20220349

• Investigator: Rebecca Hahn, MPH, 777 Canal View Blvd, Ste 1400 Rochester, New York 14623-2828 United States

This research is being overseen by WCG IRB. An IRB is a group of people who perform independent review of research studies. You may talk to them at 855-818-2289 or [researchquestions@wcgirb.com](mailto:researchquestions@wcgirb.com) if:

- You have questions, concerns, or complaints that are not being answered by the research team.
- You are not getting answers from the research team.
- You cannot reach the research team.
- You want to talk to someone else about the research.
- You have questions about your rights as a research subject.

Do you consent to these terms and wish to continue with the survey?

1. Yes [CONTINUE]
2. No [TERMINATE]

**ALL RESPONDENTS**

**S2** To begin, we would like to gather some basic information to be used for categorization purposes.

Are you…?

1 Male

2 Female

3 Other [DO NOT DISPLAY FOR INDONESIA, PAKISTAN OR MALAYSIA]

**ALL RESPONDENTS**

**S3** In what year were you born?

*Please enter as a four-digit number, e.g., 1963.*

[RANGE: 1920-2020]

|_|_|_|_|

**ALL RESPONDENTS**

**S4** HIDDEN COMPUTE FOR AGE

[IF AGE 18 OR OLDER (S4>17) ASK S5. ELSE, Terminate]

**AGE 18 OR OLDER (S4>17)**

**S5**

[FOR BANGLADESH DISPLAY: In which division is the practice where you spend most of your time located?

[INSERT DROP DOWN MENU; ALPHA SORT]

1. Barishal
2. Chittagong
3. Dhaka or Mymensingh
4. Khulna
5. Rajshaha
6. Rangpur
7. Sylhet

99. I do not practice in Bangladesh [ANCHOR] [TERMINATE]

[IF PRACTICES IN BANGLADESH (S5/NE99) CONTINUE. ELSE TERMINATE]

[FOR INDIA DISPLAY:

In which state or territory is the practice where you spend most of your time located?

[INSERT DROP DOWN MENU; ALPHA SORT]

States:

1. Andhra Pradesh
2. Arunachal Pradesh
3. Assam
4. Bihar
5. Chhattisgarh
6. Goa
7. Gujarat
8. Haryana
9. Himachal Pradesh
10. Jharkhand
11. Karnataka
12. Kerala
13. Madhya Pradesh
14. Maharashtra
15. Manipur
16. Meghalaya
17. Mizoram
18. Nagaland
19. Odisha
20. Punjab
21. Rajasthan
22. Sikkim
23. Tamil Nadu
24. Telangana
25. Tripura
26. Uttar Pradesh
27. Uttarakhand
28. West Bengal

Union Territories:

1. Andaman and Nicobar Islands
2. Chandigarh
3. Dadra & Nagar Haveli and Daman & Diu
4. Delhi
5. Jammu and Kashmir
6. Lakshadweep
7. Puducherry
8. Ladakh

99. I do not practice in India [ANCHOR] [TERMINATE]

[IF PRACTICES IN INDIA (S5/NE99) CONTINUE. ELSE TERMINATE]

**HIDDEN VARIABLE:**

**S5A** [REGIONAL RECODES INDIA]

1. Andhra Pradesh [S5=1]
2. Bihar [S5=4]
3. Gujarat [S5=7]
4. Karnataka [S5=11]
5. Madhya Pradesh [S5=13]
6. Maharashtra [S5=14]
7. Rajasthan [S5=21]
8. Tamil Nadu [S5=23]
9. Uttar Pradesh [S5=26]
10. West Bengal [S5=28]
11. All other regions [S5=2,3,5,6,8,9,10,12,15,16,17,18,19,20,22,24,25,27,29-36]

[FOR INDONESIA DISPLAY:

In which region is the practice where you spend most of your time located?

[INSERT DROP DOWN MENU; ALPHA SORT]

1. Jabodetabek
2. West Java
3. Banten
4. Central Java
5. Yogyakarta
6. East Java
7. Sumatera
8. Sulawesi
9. Kalimantan
10. Bali-Nusa Tenggara
11. Maluku-Papua

99. I do not practice in Indonesia [ANCHOR] [TERMINATE]

[IF PRACTICES IN INDONESIA (S5/NE99) CONTINUE. ELSE TERMINATE

[HIDDEN VARIABLE:

S5A [REGIONAL RECODE FOR INDONESIA]

1. Java [S5=1-6]
2. Sumatera [S5=7]
3. Sulawesi [S5=8]
4. Kalimantan [S5=9]
5. Bali-Nusa Tenggara [S5=10]
6. Maluku-Papua [S5=11]

[FOR MALAYSIA DISPLAY:

In which region is the practice where you spend most of your time located?

[INSERT DROP DOWN MENU; ALPHA SORT]

1. Selangor
2. Johor
3. Sabah
4. Sarawak
5. Perak
6. Kedah
7. W.P. Kuala Lumpur
8. Pulau Pinang
9. Kelatan
10. Pahang
11. Terengganu
12. Negeri Sembilan
13. Melaka
14. Perlis
15. W.P. Labuan
16. W.P. Putrajaya

99. I do not practice in Malaysia [ANCHOR] [TERMINATE]

[IF PRACTICES IN MALAYSIA (S5/NE99) CONTINUE. ELSE TERMINATE

[HIDDEN VARIABLE:

S5A [REGIONAL RECODES MALAYSIA]

1. Selangor [S5=1]
2. Johor [S5=2]
3. Sabah [S5=3]
4. Sarawak [S5=4]
5. Perak [S5=5]
6. Kedah [S5=6]
7. W.P. Kuala Lumpur [S5=7]
8. Pulau Pinang [S5=8]
9. Kelatan [S5=9]
10. Pahang [S5=10]
11. All other regions [S5=11]

[FOR PAKISTAN DISPLAY:

In which province or territory is the practice where you spend most of your time located?

[INSERT DROP DOWN MENU; ALPHA SORT]

1. Balochistan
2. Punjab
3. Sindh
4. Khyber Pakhtunkhwa
5. Islamabad
6. Gilgit Baltistan
7. Azad Jammu and Kashmir

99. I do not practice in Pakistan [ANCHOR] [TERMINATE]

[IF PRACTICES IN PAKISTAN (S5/NE99) CONTINUE. ELSE TERMINATE

[FOR PHILIPPINES DISPLAY:

In which region is the practice where you spend most of your time located?

[INSERT DROP DOWN MENU; ALPHA SORT]

1. Calabarzon
2. National Capital Region
3. Central Luzon Region
4. Central Visayas
5. Western Visayas
6. Bicol
7. Ilocos Region
8. Davao Region
9. Northern Mindanao
10. Soccsksargen
11. Eastern Visayas
12. Bangsamoro Autonomous Region in Muslim Mindanao (BARMM)
13. Zamboanga Peninsula
14. Cagayan Valley Region
15. Mimaropa Region
16. Caraga
17. Cordillera (CAR)

99. I do not practice in Philippines [ANCHOR] [TERMINATE]

[IF PRACTICES IN PHILIPPINES (S5/NE99) CONTINUE. ELSE TERMINATE]

[HIDDEN VARIABLE:

S5A [REGIONAL RECODES PHILIPPINES]

1. Calabarzon [S5=1]
2. National Capital [S5=2]
3. Central Luzon [S5=3]
4. Central Visayas [S5=4]
5. Western Visayas [S5=5]
6. Bicol [S5=6]
7. Ilocos [S5=7]
8. Davao [S5=8]
9. Northern Mindanao [S5=9]
10. Soccsksargen [S5=10]
11. All other regions [S5=11-17]

[FOR SINGAPORE DISPLAY:

In which region is the practice where you spend most of your time located?

[INSERT DROP DOWN MENU; ALPHA SORT]

1. North-East
2. Central
3. West
4. East
5. North

99. I do not practice in Singapore [ANCHOR] [TERMINATE]

[IF PRACTICES IN SINGAPORE (S5/NE99) CONTINUE. ELSE TERMINATE]

[FOR THAILAND DISPLAY:

In which region is the practice where you spend most of your time located?

[INSERT DROP DOWN MENU; ALPHA SORT]

1. Bangkok
2. Central Region (excluding Bangkok)
3. Northern Region
4. Northeastern Region
5. Southern Region

99. I do not practice in Thailand [ANCHOR] [TERMINATE]

[IF PRACTICES IN THAILAND (S5/NE99) CONTINUE. ELSE TERMINATE]

[FOR VIETNAM DISPLAY:

In which region is the practice where you spend most of your time located?

[INSERT DROP DOWN MENU; ALPHA SORT]

1. Northern Midlands and Mountains
2. Red River Delta
3. North and South Central Coast
4. Central Highlands
5. Southeast
6. Mekong River Delta

99. I do not practice in Vietnam [ANCHOR] [TERMINATE]

[IF PRACTICES IN VIETNAM (S5/NE99) CONTINUE. ELSE TERMINATE]

**PRACTICES IN MALAYSIA**

**S20** Which of the following best describes your ethnicity?

1. Malay
2. Chinese
3. Malay Indian
4. Other ethnicity

**PRACTICES IN TARGET COUNTRY (S5/NE99)**

**S6** Which of the following best describes your title?

1. Physician [CONTINUE]
2. Nurse [TERMINATE]
3. Pharmacist (Pharm.D.) [TERMINATE]
4. Other [ANCHOR] [TERMINATE]

[PHYSICIAN (S6/1) CONTINUE]

**PHYSICIAN (S6/1)**

**S7** What best describes your primary medical specialty?

[ALPHA SORT]

1. Family Practice
2. General Practice
3. Internal Medicine
4. Bariatric Surgery [TERMINATE]
5. General Surgery [TERMINATE]
6. Obstetrics and Gynecology
7. Endocrinology/Diabetology
8. Cardiology
9. Gastroenterology
10. Bariatrics/Obesity Medicine
11. Orthopedist
12. Aesthetics Medicine
13. Nutrition Specialist
14. Plastic Surgery [TERMINATE]
15. Other [TERMINATE]

[QUALIFY IF:

[BANGLADESH: IM, FP, GM, ENDOCRINOLOGY, CARDIOLOGY, OB/GYN, NUTRITION SPECIALIST, ORTHOPEDIST (S7/1,2, 3,6,7,8,11,13)]

[FOR INDIA: FP, GP, IM, OB/GYN, ENDOCRINOLOGY, CARDIOLOGY, GASTROENTEROLOGY, NUTRITION SPECIALIST (S7/1-3, 6-9, 13)

[INDONESIA: FP, GP, IM, ENDOCRINOLOGY, CARDIOLOGY, GASTROENTEROLOGY, OB/GYN, BARIATRICS/OBESITY MEDICINE, NUTRITION SPECIALIST (S7/1-3,6-10,13)

[MALAYSIA: FP, GP, IM, ENDOCRINOLOGY, GASTROENTEROLOGY, CARDIOLOGY, OB/GYN, NUTRITION SPECIALIST, AESTHETICS MEDICINE (S7/1-3, 6-9, 12, 13)

[PAKISTAN : FP, GP, IM, ENDOCRINOLOGY , GASTROENTEROLOGY, CARDIOLOGY, OB/GYN, NUTRITION SPECIALIST, ORTHOPAEDIST (S7/1-3, 6-9, 11, 13)

[PHILIPPINES: FP, GP, IM, ENDOCRINOLOGY, GASTROENTEROLOGY, CARDIOLOGY, OB/GYN, NUTRITION SPECIALIST (S7/1-3, 6-9, 13)

[SINGAPORE: FP, GP, IM, ENDOCRINOLOGY, GASTROENTEROLOGY, CARDIOLOGY, OB/GYN, AESTHETICS MEDICINE (S7/1-3, 6-9,12)

[THAILAND: IM, ENDOCRINOLOGY, NUTRITION SPECIALIST (S7/3,7,13)

VIETNAM: FP, IM, ENDOCRINOLOGY, CARDIOLOGY, OB/GYN, ORTHOPAEDIST, AESTHETIC PHYSICIAN (S7/1,3,6-8, 11,12)

**PHYSICIAN (S6/1)**

**S8** What percentage of your professional time is spent performing each of the following activities?

*Your best estimate will do. Your responses must sum to 100%.*

[SHOW TOTAL SUM INDICATOR; MUST TOTAL TO 100]

[RANGE 0-100]

1. Patient care/medical management I_I_I_I%
2. Surgical procedures I_I_I_I%
3. Office procedures (endoscopies, imaging, etc.) I_I_I_I%
4. Research or administrative tasks I_I_I_I%

[AT LEAST 50% TIME IN PATIENT CARE/MEDICAL MANAGEMENT (S8_1 >49) CONTINUE, OTHERWISE TERMINATE]

**SPENDS 50%+ OF TIME IN DIRECT PATIENT CARE (S8/1>49)**

**S9** How many years have you been in practice beyond your residency or fellowship]?

*If you are still in your residency, are currently a fellow, or have not been in practice for at least one year, please enter “0” (zero).*

[RANGE: 0-50]

Years in practice |_|_|

[IF IN PRACTICE 2+ YEARS (S9/2+) GO TO S9A, ELSE TERMINATE]

**IN PRACTICE 2+ YEARS (S9/2+)**

**S9A HIDDEN QUESTION FOR SOFT QUOTA**

1. Less than 5 years
2. 5 to less than 15 years
3. 15 to less than 25 years
4. 25 to less than 35 years
5. 35+ years

**IN PRACTICE 2+ YEARS (S9/2+)**

**S10** In the past month, approximately how many total adults (age 18 and older) did you personally see across all conditions and across all care settings (hospitals, outpatient clinics, etc.)?

*Your best estimate is fine.*

[RANGE: 0-9999]

Patient(s) in past month [_|_I_I_]

[IF SEEN AT LEAST 100 PATIENTS (S10>99) ASK S11. ELSE TERMINATE]

**SEEN AT LEAST 100 PATIENTS (S10>99)**

**S11** In the past month, approximately how many total adults (age 18 and older) with obesity did you personally see as defined below?

*Please consider all persons that had obesity, whether or not their weight was discussed during their visit.*

Please use the following definition for the remainder of this survey:

A **person with obesity** is:

[ALL COUNTRIES EXCEPT SINGAPORE DISPLAY: A person with a Body Mass Index (BMI) 25 or greater with or without comorbidities.]

[SINGAPORE DISPLAY: A person with a Body Mass Index (BMI) 27 or greater with or without comorbidities.]

*Your best estimate is fine.*

[RANGE: 0-S10]

Persons with obesity in past month [_|_I_I_]

[IF SEEN AT LEAST 10 PERSONS WITH OBESITY (S11>9) ASK S12. ELSE TERMINATE]

**SEEN AT LEAST 10 PERSONS WITH OBESITY (S11>9)**

**S12** Considering all of your adult patients, what percentage do you see primarily for obesity?

[RANGE 0-100]

|_|_|_|%

**ALL RESPONDENTS**

**S100 HIDDEN QUOTA QUESTIONS**

1. **QUALIFIED PCP [N=XXX]**
   - PHYSICIAN (S6/1)
   - SPECIALTY IS PCP: FAMILY PRACTICE OR GENERAL PRACTICE OR INTERNAL MEDICINE (S7/1-3)
   - SPENDS AT LEAST 50% OF TIME IN PATIENT MEDICAL MANAGEMENT (S8_1>49)
   - PRACTICES IN PARTICIPATING COUNTRY (S5/NE99)
   - IN PRACTICE 2+ YEARS (S9/2+)
   - SEEN AT LEAST 100 ADULT PATIENTS IN PAST MONTH (S10>99)
   - SEEN AT LEAST 10 ADULT PERSONS WITH OBESITY IN PAST MONTH (S11>9)
2. **QUALIFIED SPECIALIST [N=XXX]**
   - PHYSICIAN (S6/1)
   - SPECIALTY IS OBSTETRICS/GYNECOLOGY, ENDOCRINOLOGY, ORTHOPAEDIST, CARDIOLOGY, GASTROENTEROLOGY, NUTRITION SPECIALIST, BARIATRIC/OBESITY MEDICINE, AESTHETICS MEDICINE (S7 6-13)]
   - SPENDS AT LEAST 50% OF TIME IN PATIENT MEDICAL MANAGEMENT (S8_1>49)
   - PRACTICES IN PARTICIPATING COUNTRY (S5/NE99)
   - IN PRACTICE 2+ YEARS (S9/2+)
   - SEEN AT LEAST 100 PATIENTS IN PAST MONTH (S10>99)
   - SEEN AT LEAST 10 PERSONS WITH OBESITY IN PAST MONTH (S11>9)
3. **UNQUALIFIED HCPS [N=9999]**

**ALL RESPONDENTS**

**S105 SOFT QUOTA FOR OBESITY SPECIALISTS**

1. **OBESITY SPECIALIST [N=9999**
   - QUALIFIED HCP (S100/1-2)
   - 50% OR MORE PATIENTS SEEN FOR OBESITY/WEIGHT MANAGEMENT (S12/>49%)

**ALL RESPONDENTS**

**S110 SPECIALTY SOFT QUOTAS**

| **Specialty** | **Indonesia** | **India** | **Pakistan** | **Thailand** | **Malaysia** | **Singapore** | **Philippines** | **Vietnam** | **Bangladesh** |
| --- | --- | --- | --- | --- | --- | --- | --- | --- | --- |
| Family Practice | Max n=50 | Max n=50 | Max n=50 |  | Max n=50 | Max n=50 | Max n=50 | Max n=75 | Max n=50 |
| General Practice | Max n=50 | Max n=50 | Max n=50 |  | Max n=50 | Max n=50 | Max n=50 |  | Max n=50 |
| Internal Medicine | Max n=50 | Max n=50 | Max n=50 | Max=100 | Max n=50 | Max n=50 | Max n=50 | Max n=75 | Max=100 |
| Aesthetics Medicine |  |  |  |  |  | Max=10 |  |  |  |
| **PCP Total** | **n=100** | **n=150** | **n=100** | **n=100** | **n=100** | **n=100** | **n=100** | **n=100** | **n=100** |
| Endocrinologist | Max n=30 | Max n=40 | Max n=30 | Max n=80 | Max n=30 | Max n=35 | Max n=30 | Max n=35 | Max n=35 |
| Cardiologist | Max n=30 | Max n=40 | Max n=30 |  | Max n=30 | Max n=35 | Max n=30 | Max n=35 | Max n=35 |
| Gastroenterologist | Max n=30 | Max n=40 | Max n=30 |  | Max n=30 | Max n=35 | Max n=30 |  |  |
| Ob/Gyn | Max n=15 | Max n=20 | Max n=15 |  | Max n=15 | Max n=20 | Max n=20 | Max n=20 | Max n=20 |
| Nutrition Specialist (MD) | Max n=15 | Max n=20 | Max n=15 | Max n=50 | Max n=15 |  | Max n=20 |  | Max n=20 |
| Bariatrics/Obesity Medicine | Max n=15 |  |  |  |  |  |  |  |  |
| Orthopedist |  |  | Max n=15 |  |  |  |  | Max n=20 | Max n=20 |
| Aesthetic Physician |  |  |  |  | Max n=15 | Max=10 |  | Max n=20 |  |
| **Specialist Total** | **n=100** | **n=150** | **n=100** | **n=100** | **n=100** | **n=100** | **n=100** | **n=100** | **n=100** |
| **Total HCPs** | **n=200** | **n=300** | **n=200** | **n=200** | **n=200** | **n=200** | **n=200** | **n=200** | **n=200** |

**SECTION 100: Patient Weight History / Demographics**

**ALL RESPONDENTS**

**Q100** Thank you for your responses. You have qualified for this research. Considering your entire patient population, what proportion of your patients fall into each of the following groups? If none fit into a category, please enter “0” (zero).

*Your best estimate will do. Your responses should sum to 100%.*

[INSERT CONSTANT SUM INDICATOR. TOTAL MUST SUM TO 100]

[RANGE 0-100]

[FOR ALL COUNTRIES EXCEPT SINGAPORE DISPLAY:

1. Not overweight (BMI < 25) |_|_|_| %
2. Obesity class 1 (BMI 25 to 29.9) |_|_|_| %
3. Obesity class 2 (BMI 30 to 34.9) |_|_|_| %
4. Obesity class 3 (BMI 35 to 39.9) |_|_|_| %
5. Obesity class 4 (BMI 40+) |_|_|_| %

[FOR SINGAPORE DISPLAY:

1. Not overweight (BMI < 27) |_|_|_| %
2. Obesity class 1 (BMI 27 to 31.9) |_|_|_| %
3. Obesity class 2 (BMI 32 to 36.9) |_|_|_| %
4. Obesity class 3 (BMI 37 to 41.9) |_|_|_| %
5. Obesity class 4 (BMI 42+) |_|_|_| %

**SECTION 200: READINESS TO CHANGE / PREVIOUS SUCCESS**

PwO: Q205

**ALL RESPONDENTS**

**Q215** As a reminder, throughout this survey, we are defining persons with obesity as those with a Body Mass Index (BMI) of [FOR ALL COUNTRIES EXCEPT SINGAPORE DISPLAY:

“25” [FOR SINGAPORE DISPLAY: “27”] or greater with or without comorbidities.

To the best of your knowledge, what percentage of your patients with obesity have made what you consider a serious attempt to lose weight (e.g., followed a program, set goals, put their mind to it, or worked with a qualified healthcare professional), whether or not it was successful?

*Please provide your best estimate.*

[RANGE 0-100]

Percentage of patients with obesity that have made a serious attempt to lose weight |_|_|_|%

**ALL RESPONDENTS**

**Q217** In general, what percentage of your patients who made a serious attempt to lose weight within the past year would you define as successful?

*Your best estimate will do.*

[RANGE 0-100]

Percentage of patients |_|_|_|%

PwO: Q203

**ALL RESPONDENTS**

**Q225** In your experience, which of the following most motivates people to lose weight?

*Select all that apply.*

[MULTIPLE RESPONSE, RANDOMIZE WITHIN GROUPS. RANDOMIZE GROUP ORDER, SELECT ALL THAT APPLY], don’t SHOW HEADINGS]

**Physical Health**

1. Having general health concerns
2. Wanting to stop or not need to take medication for a weight-related health condition
3. Reaching the upper end of the weight range they are comfortable with
4. A specific, personal medical event (heart attack, stroke, etc.) or diagnosis (diabetes, liver disease, sleep apnea, etc.)

**Support**

1. Encouragement, support, recommendations from family or friends
2. Encouragement, support, recommendations from a healthcare provider
3. Encouragement, support, recommendations from wellness / fitness programs or a personal trainer
4. Encouragement, support from others who are trying to lose weight (losing weight with a spouse, health or fitness competition, etc.)

**Appearance**

1. Wanting to fit into a smaller clothing size
2. Wanting to be more fit / in better shape

**Goals**

1. Wanting to feel better physically, have more energy or be more active
2. Wanting to be more confident / improve their self-esteem
3. Wanting to improve job performance
4. Wanting to improve their sex life
5. Wanting to be a positive role model for family / children

**Life Events**

1. A major life change such as retirement, divorce, break-up, starting a family
2. An upcoming special occasion or event
3. A specific medical event (heart attack, stroke, etc.) or diagnosis (diabetes, liver disease, sleep apnea, etc.) in a family member / close friend
4. Other [ANCHOR]
5. None of the above [EXCLUSIVE, ANCHOR]

**ALL RESPONDENTS**

**Q230** How do you monitor the effectiveness of a weight management strategy or treatment for your patients with obesity?

*Please select all that apply.*

[RANDOMIZE]

1. Amount of weight loss by % body weight
2. Reduction in BMI
3. Reduction in waist circumference
4. Decrease or cessation of patient’s weight loss efforts
5. Changes/improvements in patient’s quality of life
6. Patient feedback / satisfaction
7. Patient adherence to lifestyle modifications
8. Changes/improvements in patient’s comorbidities
9. Comparing patient results against obesity management guidelines / algorithms
10. Amount of weight loss in kilograms
11. Prevention of further weight gain or weight stabilization
12. Amount of visceral fat loss measured by CT or MRI
13. I do not monitor effectiveness [ANCHOR, EXCLUSIVE]

**ALL RESPONDENTS**

**Q235A** For what proportion of your patients with obesity do you actually discuss their weight?

[RANGE 0-100]

|_|_|_|% of patients with obesity with whom I discuss their weight

**ALL RESPONDENTS**

**Q240** For quality control purposes, select “Completely agree” from the list of options below.

1. Completely disagree
2. Somewhat disagree
3. Neutral
4. Somewhat agree
5. Completely agree

**SECTION 400: OBESITY AWARENESS AND PERCEPTIONS**

PwO: Q405

**ALL RESPONDENTS**

**Q405** In general, how large of an impact do you believe the following health conditions have on a person’s overall health?

*Use a scale where 1 means “Very little impact” and 5 means “An extreme impact.”*

*1 - Very little impact 2 3 4 5 - An extreme impact*

[RANDOMIZE]

1. Diabetes
2. Chronic obstructive pulmonary disease (COPD)
3. Cancer
4. Stroke
5. Obesity [FOR ALL COUNTRIES EXCEPT SINGAPORE DISPLAY: (BMI of 25 or greater)] [FOR SINGAPORE DISPLAY: (BMI of 27 or greater)]

**ALL RESPONDENTS**

**Q405A HIDDEN QUESTION**

[RECODE Q405 ATTRIBUTES 1-4 BASED ON IF THEY’RE GREATER OR LESS THAN OBESITY (Q405_5)]

[COLUMNS]

OBESITY IS MORE SERIOUS (Q405_ATTRIBUTE < Q405_5)

OBESITY IS AS SERIOUS (Q405_ATTRIBUTE = Q405_5)

OBESITY IS LESS SERIOUS (Q405_ATTRIBUTE > Q405_5)

**ALL RESPONDENTS**

**Q410** Compared to a person who does not have obesity, how easy or difficult do you think each of the following is for someone who has obesity?

| [RANDOMIZE] | Much harder | Somewhat harder | About the same | Somewhat easier | Much easier |
| --- | --- | --- | --- | --- | --- |
| Getting a job | 1 | 2 | 3 | 4 | 5 |
| Advancing/promotion in a job | 1 | 2 | 3 | 4 | 5 |
| Making friends | 1 | 2 | 3 | 4 | 5 |
| Forming romantic relationships | 1 | 2 | 3 | 4 | 5 |

**ALL RESPONDENTS**

**Q415** How much of an impact do you think a person having obesity has on each of the following?

| [RANDOMIZE] | Very negative impact | Somewhat negative impact | No impact | Somewhat positive impact | Very positive impact |
| --- | --- | --- | --- | --- | --- |
| How smart people think the person is | 1 | 2 | 3 | 4 | 5 |
| How athletic people think the person is | 1 | 2 | 3 | 4 | 5 |
| How healthy people think the person is | 1 | 2 | 3 | 4 | 5 |
| Relationships with friends | 1 | 2 | 3 | 4 | 5 |
| Relationships at home/with family | 1 | 2 | 3 | 4 | 5 |
| How much ambition people think the person has | 1 | 2 | 3 | 4 | 5 |
| How much willpower people think the person has | 1 | 2 | 3 | 4 | 5 |

**SECTION 500: OBESITY ATTITUDINAL QUESTIONS**

PwO: Q500

**ALL RESPONDENTS**

**Q503** Thinking of your patients with obesity as a whole, please indicate how much you agree with each of the following…

*Use a scale where 1 means “Do not agree at all” and 5 means “Completely agree.”*

*1 - Do not agree at all 2 3 4 5 - Completely agree*

[RANDOMIZE,CAROUSEL]

1. It is easy for my patients to lose weight.
2. My patients could lose weight if they really set their mind to it.
3. If my patients lost weight, it would be easy for them to keep the weight off.
4. My patients know what they need to do to lose weight.
5. I have a responsibility to actively contribute to my patients’ weight loss effort.
6. My patients’ weight loss is completely their responsibility.
7. For my patients to lose weight, they would need to completely change their lifestyles.
8. My patients are happy with their current weight.
9. My patients are past the point where they can lose weight on their own.
10. My patients are motivated to lose weight.
11. For quality control purposes, please select 1.
12. Obesity is less important than many of the other diseases I treat.
13. I do not feel comfortable bringing up a patient’s weight unless they mention it first.
14. There is nothing I can do to help patients manage their weight.
15. Treating patients with obesity is a productive use of my time.
16. I feel motivated to help patients with obesity lose weight.
17. I support/empower my patients with obesity to make healthy changes.
18. My patients know how to keep the weight off.

PwO: Q507

**ALL RESPONDENTS**

**Q507** How much do you agree that each of the following is a barrier to your patients losing weight?

*Please use a scale where 1 means “Do not agree at all” and 5 means “Completely agree.”*

1 - Do not agree at all 2 3 4 5 - Completely agree

[RANDOMIZE, CAROUSEL]

1. Their preference for unhealthy food
2. Lack of exercise
3. Their genes
4. The nature of their job / employment
5. A lack of time to cook healthy meals
6. Their other health conditions
7. Their friends and family
8. Me, their healthcare provider
9. Their finances
10. Their lack of motivation
11. Their lack of ability to control their hunger
12. The cost of healthy food
13. Limited access to healthy food
14. Their mental health/emotional state
15. Fear of failure
16. Limited coverage for health care costs
17. Limited mobility due to physical health problems
18. The possibility of regaining the weight
19. Their unhealthy eating habits (large portions sizes, excessive snacking)
20. Their lack of understanding of what obesity is
21. The cost of obesity medications, programs and services
22. Their metabolism
23. Their age
24. High carbohydrate diet

Answer list is same as Q210A in PwO (methods discussed) and repeats in HCP at 515

**ALL QUALIFIED**

**Q128** Still thinking of your patients with obesity, for what percentage do you **recommend** each of the following methods for weight management when discussing their weight (whether or not the patient followed your recommendation)?

*Your responses may sum to more than 100% to account for instances where you may make more than one treatment recommendation.*

[RANGE 0-100, RANDOMIZE ROWS WITHIN GROUP, RANDOMIZE GROUP ORDER EXCEPT 16 AND 99 ITEMS, DON’T SHOW HEADINGS] [NUMERIC BOX NEXT TO EACH ROW ITEM]

[MAKE NONE OF THE ABOVE EXCLUSIVE] [ANCHOR] [MAY SUM TO MORE OR LESS THAN 100%]

**Diet / Healthy Eating**

1. General improvement in eating habits / reducing calories
2. Specific diet or diet program [FOR INDONESIA DISPLAY: (e.g. Keto diet, intermittent fasting, OCD diet, Mayo diet, high protein diet, Mediterranean diet, mesotherapy)] [FOR INDIA DISPLAY: (e.g. Keto diet, intermittent fasting, vegan diet, low carb)] [FOR PAKISTAN DISPLAY: (e.g. Keto diet, Atkins, Mediterranean diet)] [FOR MALAYSIA DISPLAY: (e.g. plate method, meal replacement program, low glycemic index, moderate carb diet)] [FOR SINGAPORE DISPLAY: (e.g. Low-carb diet, Keto diet, intermittent fasting, fixed caloric deficit, very low calorie or low calorie diet with meal replacements, time-restricted feeding, low-fat diet)] [FOR PHILIPPINES DISPLAY: (e.g. Calorie Counting, Intermittent Fasting, Vegan/Vegetarian, Paleo, Ketogenic diet, low-fat, South Beach diet, gluten free diet, Blood type diet)] [FOR THAILAND, VIETNAM AND BANGLADESH DISPLAY: (e.g. Keto, low-carb, low-fat, intermittent fasting)]
3. Elimination diets (avoiding fats, sugary beverages, carbohydrates, etc.)

**Exercise**

1. Generally be more active / increase physical activity
2. A formal exercise program / Gym membership / Personal trainer

**Tracking**

1. Meal / nutrient tracking (on paper or an app)
2. Exercise tracking (on paper or app such as smartphone apps, wearable fitness tracker, etc.)

**Medical Treatment / Medication**

1. Over-the-counter (non-prescription) weight loss medication (vitamins, supplements, etc.)
2. Prescription weight loss medication
3. Visiting a nutritionist / dietitian (non-physician)
4. Visiting an obesity specialist
5. Behavior therapy or psychotherapy such as counseling or behavior modification
6. Weight loss surgery / bariatric surgery

**Quality of life management**

1. Stress management
2. Sleep quality management
3. Other [ANCHOR]
4. None of the above [EXCLUSIVE][ANCHOR]

PwO: Q210D

**ALL QUALIFIED**

**Q515** Which of the following do you believe are **most effective for long-term weight management**?

*Please select all that apply.*

[MULTI SELECT] [INSERT ENTIRE LIST FROM Q128. SHOW IN SAME ORDER AS Q128]

PwO: Q520

**ALL RESPONDENTS**

**Q521** Please indicate how much you agree with the following regarding prescription medications for weight loss.

*Use a scale where 1 means “Do not agree at all” and 5 means “Completely agree.”*

[RANDOMIZE, CAROUSEL]

1 - Do not agree at all 2 3 4 5 - Completely agree 99 Don’t know/ Not sure

1. Prescription weight loss medications are more effective for my patients than other treatment options for weight loss.
2. I am likely to prescribe new prescription weight loss medications.
3. I am more likely to recommend my patients take a prescription medication than have a surgery (bariatric) to lose weight.
4. I am concerned about the side effects associated with prescription weight loss medications.
5. My patients would like me to offer prescription weight loss medication to help them with their weight loss efforts.
6. My patients trust me to recommend a prescription weight loss medication that is right for them.
7. There are good options available today for prescription weight loss medications.
8. Cost is a major barrier for my patients to consider prescription weight loss medications.
9. Patients would rather lose weight on their own than depend on medication.
10. I am likely to review the prescription weight loss medications available with my patients.
11. I am concerned about the long-term safety associated with prescription weight loss medications.
12. I don’t know enough about prescription weight loss medications to feel comfortable prescribing them to my patients with obesity.
13. I tend to use anti-obesity medications for the short term (e.g. 6 months or less)

PwO: Q525

**ALL RESPONDENTS**

**Q525** Please indicate how much you agree with the following regarding weight loss surgery.

*Weight loss surgery is also known as bariatric surgery.*

*Use a scale where 1 means “Do not agree at all” and 5 means “Completely agree.”*

[RANDOMIZE, CAROUSEL]

1 - Do not agree at all 2 3 4 5 - Completely agree 99 Don’t know/ Not sure

1. Weight loss surgery is more effective than other treatment options for weight loss.
2. Most of my patients would rather have weight loss surgery than change their lifestyle to lose weight.
3. My patients often have concerns about the safety of weight loss surgery.
4. My patients trust me to recommend a weight loss surgery if it’s right for them.
5. There are good options available today for weight loss surgery.
6. Cost is a major barrier for my patients considering weight loss surgery.
7. I am likely to review the weight loss surgery options with my patients.
8. The wait time for surgery is too long.
9. Having weight loss surgery also means having a permanent change in lifestyle.
10. I would rather motivate my patients to lose weight with diet and exercise than recommend weight loss surgery.
11. Having weight loss surgery is the easy way out for the patient.
12. Weight loss after surgery is often not maintainable by patients.
13. Weight loss surgery is a last option after patients have failed attempts with lifestyle and anti-obesity medications.

**SECTION 600: SUPPORT STRUCTURE**

PwO: Q826

**ALL RESPONDENTS**

**Q602** What are the **top 5 types of support that would be most helpful** for your patients to be successful with managing their weight?

*Select your top 5. [MUST SELECT 5]*

[5 SELECTIONS] [RANDOMIZE]

1. Resources for family and friends to help understand how to be supportive
2. Specific meal plans to follow for weight management
3. Online support groups for those trying to lose weight
4. Local in-person support groups for those trying to lose weight
5. Motivational programs to help people stay on track with weight loss plan
6. More programs offered at workplaces to help people lose weight
7. Encouragement from friends/family to increase desire to keep going
8. Financial support for healthy choices (gym membership, healthy foods)
9. Diary for tracking weight over time (paper based or electronic)
10. Diary for tracking food intake (paper based or electronic)
11. Diary for tracking physical activity (paper based or electronic)
12. App with weight loss tracking and ideas for healthy eating and physical activity
13. Programs for physical activity
14. Prescription drugs for weight loss
15. Over-the-counter drugs for weight loss
16. Personal trainer / weight loss counselor
17. Weekly follow-up with a healthcare provider
18. Meetings with dietitian / nutritionist (non-physician)
19. A work culture that encourages a healthy lifestyle
20. Access to mental health support
21. Access to stress management support
22. Access to a physician who specializes in obesity
23. Other [ANCHOR]

PwO: Q860

**ALL RESPONDENTS**

**Q605** Which of the following do you think contribute to a person’s successful weight loss efforts?

*Please select all that apply.*

[MULTISELECT][RANDOMIZE]

1. Setting realistic goals which seem possible to reach
2. Support from family/friends
3. Support from me or other health care providers
4. Wanting to reduce the negative health consequences of their weight
5. Wanting to be happier with the way they look
6. Wanting to be able to fit into their clothes
7. Wanting to reduce emotional problems associated with their weight
8. Wanting to reduce physical limitations caused by their weight
9. Wanting to improve intimacy (be more appealing/sexually confident)
10. A desire to improve their job performance
11. Using prescription weight loss medications
12. Having the motivation or determination to lose weight
13. Adherence/sticking to their regimen or weight loss plan
14. Being energized by their success
15. Prioritizing their weight loss activities into their daily life
16. Understanding the physiology of obesity
17. A customized weight loss plan
18. Other [ANCHOR]
19. None of these [ANCHOR, EXCLUSIVE]

PwO: Q640

**ALL RESPONDENTS**

**Q650** Please indicate how much you agree with the following statements regarding obesity and weight management:

*Use a scale where 1 means “Do not agree at all” and 5 means “Completely agree.”*

1 - Do not agree at all 2 3 4 5 - Completely agree 99 - Does not apply

[RANDOMIZE]

1. Maintaining a healthy weight is a priority for our country’s healthcare system.
2. Cost of obesity therapy / treatment is a barrier for patients to lose weight.
3. I feel the healthcare system (doctor’s offices, hospitals, etc.) is a good resource for those looking to lose weight.
4. Employers play an important role in managing patients’ weight.
5. Obesity is a chronic disease.
6. A loss of 5-10% body weight would be extremely beneficial to the overall health of a patient with obesity.
7. The treatment of obesity should be a team effort between different medical professionals.
8. For quality control purposes, please select 3.

**SECTION 700: INTERACTION WITH PATIENTS**

PwO: Q701/Q702

**ALL RESPONDENTS**

**Q702** When discussing obesity with a patient, what percentage of the time do you bring it up, and what percentage of the time does the patient bring it up?

[INSERT CONSTANT SUM INDICATOR. TOTAL MUST SUM TO 100]

[RANGE 0-100]

1. I start the conversation |_|_|_|%
2. The patient starts the conversation |_|_|_|%

**ALL RESPONDENTS**

**Q704** What are the top-5 most important criteria that you consider in order to determine whether or not you will initiate a discussion with a patient about obesity?

*Please select only 5 items. [MUST SELECT 5]*

[MULTISELECT, RANDOMIZE]

1. Patient’s BMI
2. Patient’s weight
3. Patient’s vital signs (i.e., blood pressure, heart rate)
4. Patient is at risk of developing new/additional obesity-related comorbidities
5. Patient has obesity-related comorbidities
6. Patient will need to take medication for obesity-related comorbidities if they do not lose weight
7. Patient’s weight has increased since previous appointment(s)
8. My relationship with the patient
9. How receptive I think the patient will be to discussing their weight
10. Financial considerations for my practice (able to bill for obesity diagnosis and treatment)
11. How much time I have for the visit
12. Patient’s mental state
13. Other [ANCHOR]
14. Patient’s waist circumference

**ALL RESPONDENTS**

**Q705** How comfortable are you in having discussions with your patients about their weight?

1. Not at all comfortable
2. A little comfortable
3. Somewhat comfortable
4. Very comfortable
5. Extremely comfortable

PwO: Q770

**ALL RESPONDENTS**

**Q708** What are the **top 5** reasons for which you **might not** discuss obesity with a patient?

*Please select up to 5 items only*

[MULTISELECT 5 ANSWERS, RANDOMIZE]

1. The appointment is not long enough / I’m rushed
2. There are more important health issues/concerns to discuss
3. I do not feel comfortable bringing it up
4. I do not trust and/or do not have a close relationship with my patient
5. I do not see weight as a significant medical issue
6. Patient is in good health and does not have weight-related comorbidities
7. I believe it is the patient’s responsibility to manage their weight
8. Patient is not interested in losing weight
9. Patient does not feel motivated to lose weight
10. Patient does not believe he/she is able to lose weight
11. Even if the patient were to lose weight, he/she would just gain it back
12. Patient already knows what he/she needs to do to manage their weight
13. There is nothing I can do to help patients managing their weight
14. I am not interested enough in/concerned enough about patients’ weight
15. I have had previous bad experience discussing weight with a patient
16. Patient does not have financial means to support a weight loss effort
17. I do not have training to provide weight management services
18. My office is not set up to treat overweight patients
19. I do not get financial compensation for treating obesity
20. Other [ANCHOR]

**ALL RESPONDENTS**

**Q720** What types of weight management goals do you set with your patients with obesity?

*Please select all that apply.*

[MULTISELECT] [RANDOMIZE]

1. Maintain current weight without gaining more
2. To lose (any amount of) weight
3. To lose a pre-specified % of body weight
4. To lose a pre-specified number of kilograms
5. To decrease the number of medications they must take
6. To improve their existing health condition(s)
7. To reduce the risks associated with weight / prevent a health condition
8. To have more energy
9. To improve their appearance
10. Short-term (within the next six months) individual weight loss goals
11. Long-term (more than six months from now) individual weight loss goals
12. To improve their lifestyle
13. To reduce their stress and improve overall health and well-being
14. To improve their physical and mental health and well-being
15. Other
16. I do not set weight management goals with my patients. [EXCLUSIVE]

**ALL RESPONDENTS**

**Q734** Is an obesity diagnosis typically recorded in the patient’s medical record/journal?

1. Never
2. Rarely
3. Sometimes
4. Most of the time
5. Always

**ALL RESPONDENTS**

**Q734B** In what proportion of your patients with obesity do you inform them that they have a diagnosis of obesity?

[RANGE 1-100]

Inform the patient about the diagnosis of obesity  |_|_|_|%

[ ] I never inform the patient about the diagnosis of obesity  [EXCLUSIVE]

PwO: Q759

**ALL RESPONDENTS**

**Q740A** For what proportion of your patients with obesity do you schedule a **follow-up appointment** to discuss their weight?

*Please enter ‘0’ if you don’t schedule follow up appointments to discuss their weight.*

[RANGE 0-100]

|_|_|_|% of patients with obesity for whom I schedule a **follow-up appointment** to discuss their weight

**ALL RESPONDENTS**

**Q750** For which of the following reasons might you refer a patient with obesity for specialized obesity management?

*Please select all that apply.*

[MULTISELECT, RANDOMIZE]

1. The patient asks to see a specialist
2. The patient had been unable to achieve their weight loss goals under previous treatment plan
3. The patient’s weight is related to another health condition they have
4. I do not feel confident in providing the patient the advice they need to be successful
5. They have been unsuccessful in achieving their goals under my advice
6. Coverage of weight-management related care (e.g. psychologist, dietitian, non-physician nutritionist, etc.)
7. Their financial status
8. I do not feel comfortable discussing the patient’s weight
9. Initiation of pharmacotherapy
10. Indication for bariatric surgery
11. Availability of a specialized weight loss program
12. Other [ANCHOR]
13. I do not refer patients to obesity specialists [EXCLUSIVE, ANCHOR]

**ALL QUALIFIED**

**Q759D** Which of the following clinical treatment guidelines do you follow for treating patients with obesity?

*Please select all that apply.*

1. Local
2. National
3. International (e.g., European Association for the Study of Obesity (EASO), American Heart Association / American College of Cardiology / Obesity Society Clinical Practice Guideline)
4. I do not follow clinical treatment guidelines when treating patients with obesity [EXCLUSIVE]

**ALL RESPONDENTS**

**Q758** How effective do you think current clinical guidelines for treating obesity are?

[DISPLAY AS GRID]

Local National International

1. Not at all effective
2. A little effective
3. Somewhat effective
4. Very effective
5. Extremely effective
6. Not applicable

PwO : q825

**ALL RESPONDENTS**

**Q760** Please select the top 3 most helpful types of information you can provide patients to support their weight loss efforts?

*Please select only three items.*

**Information on…**

[SELECT 3 ONLY]

1. Healthy ways to lose weight
2. The health benefits of weight loss
3. Medical treatment options for obesity
4. Managing weight with exercise
5. How to maintain weight loss
6. How healthcare providers can help with obesity
7. Stress management techniques
8. Realistic weight loss goals
9. Exercises which are safe to do for people with mobility limitations
10. Healthy vs. non-healthy eating

**SECTION 900: DEMOGRAPHICS**

**ALL RESPONDENTS**

**Q900** Thank you again for your time so far. As we noted at the beginning of this survey, your personal information will never be shared with other organizations for any purpose. Your honest answers are very much appreciated. Your responses to this survey will help the sponsor (Novo Nordisk) understand the healthcare experiences of patients.

To finish, we would like to gather some additional information used for categorization purposes.

PwO: Q900

**ALL RESPONDENTS**

**Q906** What percentage of your patients with obesity are diagnosed with each of the following conditions?

*Your best estimate will do.*

[RANGE 0-100]

[RANDOMIZE]

*|_|_|_|*%

1. Cardiovascular Diseases (e.g., coronary heart disease, congestive heart failure, atrial fibrillation, pulmonary embolism, stroke)
2. Depression/Anxiety
3. High cholesterol (Dyslipidemia / triglycerides)
4. High blood pressure (Hypertension)
5. Infertility
6. Liver disease (e.g., non-alcoholic fatty liver disease)
7. Obstructive Sleep Apnea
8. Osteoarthritis
9. Metabolic syndrome
10. Stomach or intestinal problems
11. Pre-diabetes
12. Diabetes (Type II)
13. [REMOVED]
14. Cancer
15. Polycystic Ovary Syndrome (PCOS) (the % of your *female* patients with obesity)
16. Eating disorder (e.g., binge eating disorder, night eating syndrome)

**ALL RESPONDENTS**

**Q910** Which of the following best describes your primary practice setting? If you practice in more than one location, please select the option that represents where you spend the majority of your time. Please select one option only.

[DISPLAY FOR ALL COUNTRIES EXCEPT INDIA, PAKISTAN, MALAYSIA, SINGAPORE]

1. Individual medical practice
2. Group practice (privately-owned but with shared facilities)
3. Group practice (employed)
4. Hospital (public or private)
5. Other [anchor]

[FOR INDIA DISPLAY:

1. Privately owned medical practice
2. Government health center (primary health center)
3. Hospital (not emergency department)
4. Other [ANCHOR]

[FOR PAKISTAN DISPLAY:

1. Private medical practice
2. Government hospital
3. Private hospital
4. Privately-owned medical practice
5. Other [ANCHOR]

[FOR MALAYSIA DISPLAY:

1. Privately-owned medical practice (including private hospital-owned office and clinics)
2. Group practice (privately-owned but with shared facilities)
3. Group practice (employed)
4. Hospital employed (Government, university or private)
5. Free-lance General Practitioner (Locum)
6. Other [ANCHOR]

[FOR SINGAPORE DISPLAY:

1. Polyclinic
2. Primary Care Practice with mostly self-paying patients
3. Primary Care Practice in managed healthcare (patients mainly on Corporate Health Insurance Plans)
4. Public Health Institution (PHI)
5. Private Specialist Practice
6. Other

**ALL QUALIFIED PHYSICIANS (S100/1,2)**

**Q922** Do you consider yourself an expert in obesity?

1. Yes
2. No

**ALL RESPONDENTS**

**Q925** Have you received advanced training specifically in obesity/weight management [DISPLAY FOR PHYSICIANS (S6/1): beyond medical school]?

1. Yes
2. No

**ALL RESPONDENTS**

**Q930** Do you provide care for obesity/weight management as a primary treatment objective?

1. Yes
2. No

**ALL RESPONDENTS**

**Q932** For how many years have you been providing obesity care/management to patients?

*Your best estimate will do. If less than one year, please enter “0” (zero).*

[RANGE: 0-S9]

Years providing obesity care/management to patients |_|_|

**ALL RESPONDENTS**

**Q950** Are you part of an interdisciplinary obesity treatment team?

*An interdisciplinary team may include a primary care physician, obesity specialist, surgeon, dietician, psychologist, movement therapist, and/or nurse.*

1. Yes
2. No

PwO: Q929

**ALL RESPONDENTS**

**Q920** Which one of the following best describes the community in which your primary practice is located?

1. Urban area
2. Suburban area close to a city
3. Rural area / village
