# Supplementary material for: Perceptions, attitudes, behaviours and barriers towards obesity among people with obesity and health care professionals in Indonesia: An exploratory online survey
Source: PLoS One. 2026 Jun 4;21(6):e0350857. doi: 10.1371/journal.pone.0350857 (PMC13235876; doi:10.1371/journal.pone.0350857)
Supplement: S3 File — (DOCX) [file pone.0350857.s006.docx]

We partnered with vendor for translations. They have native-speaking translators who translate documents. There were a series of checks and back-checks, then they also reviewed the translated program itself (i.e., how it presents on-screen for respondents). Regarding data validation, three data validation questions were included with simple and obvious answers to detect respondents entering fraudulent data. Respondents failing to provide the correct response to 2 or more of these questions were excluded from the survey and dataset. The final dataset was reviewed for respondents with extremely low interview times and “straight-lining” of grid questions. Respondents appearing to provide fraudulent data were reviewed on a case-by-case basis and removed from the final dataset where appropriate.
